# Contrast-sparing CT using renal-clearable gold nanoclusters for early spatial mapping of renal dysfunction

**DOI:** 10.1126/sciadv.aec0972

**Published:** 2026-05-29

**Authors:** Jinbin Pan, Li Ma, Hua Ma, Yuanlin Zhang, Iris. Y. Zhou, Nicholas J. Rotile, Dong Li, Onofrio A. Catalano, Shao-Kai Sun, Peter Caravan

**Affiliations:** ^1^ Department of Radiology, Tianjin Key Lab of Functional Imaging and Tianjin Institute of Radiology, Tianjin Medical University General Hospital, Tianjin 300052, China.; ^2^ Athinoula A. Martinos Center for Biomedical Imaging, Massachusetts General Hospital, Charlestown, MA 02129, USA.; ^3^ Institute for Innovation in Imaging, Department of Radiology, Massachusetts General Hospital, Charlestown, MA 02129, USA.; ^4^ Harvard Medical School, Boston, MA 02115, USA.; ^5^ Division of Abdominal Imaging, Department of Radiology, Massachusetts General Hospital, Boston, MA 02114, USA.; ^6^ School of Medical Imaging, Division of Medical Technology, Tianjin Key Laboratory of Functional Imaging, Tianjin Medical University, Tianjin 300203, China.

## Abstract

Early, noninvasive, and spatially resolved assessment of renal dysfunction is essential for theranostics of kidney diseases, yet clinical biomarkers are criticized for averaging whole-kidney function and missing focal or unilateral injury. Here, we present a contrast-sparing computed tomography (CT) approach that maps renal dysfunction at the compartment level using renal-clearable glutathione-gold nanoclusters (GSH-Au NCs). The merits of ultrasmall hydrodynamic size (2.8 nanometers) and high x-ray attenuation ability enable GSH-Au NCs with fast renal clearance and robust intrarenal CT contrast. In three mechanistically distinct mouse models of acute kidney injury, low-dose GSH-Au NCs produced mechanism-specific imaging signatures and distinct time-attenuation behaviors, even before biomarkers changed. Notably, at an ultralow dose (32.5 milligrams of Au per kilogram), early bilateral cisplatin injury can be easily detected with an obvious bright band at the outer medulla area on CT, consistent with tubular injury and a cast/debris-mediated mechanism. This work establishes a dose-efficient CT workflow for early, noninvasive, spatial mapping of renal dysfunction, supporting diagnosis, classification, and monitoring of kidney disease.

## Teaser

Ultralow-dose gold nanoclusters enable CT identification and localization of early kidney damage.

## INTRODUCTION

Kidney diseases impose a major global health burden (*1*). Timely recognition of renal dysfunction is pivotal to preventing irreversible loss of function and downstream complications (*2*). However, early diagnosis remains difficult because routine biochemical indicators like serum creatinine, blood urea nitrogen, and creatinine-based estimated glomerular filtration rate (eGFR) change mainly after substantial loss of functional nephron mass, show a poorly linear relationship with true GFR, and are easily influenced by muscle mass, hydration, and medications (*3*–*5*). Moreover, eGFR equations assume steady state and are unreliable in evolving injury (*6*). Critically, these markers integrate whole-kidney filtration and are therefore blind to regional heterogeneity: Unilateral or segmental dysfunction (e.g., local ischemia, early tubular injury, or unilateral outflow obstruction) may be masked by contralateral compensation and remain “normal” at the population level (*7*). Even the emerging biomarkers of early glomerular or tubular injury [e.g., serum cystatin C, β2-microglobulin, *N*-acetyl-β-d-glucosaminidase, and kidney injury molecule-1 (KIM-1)] with more sensitivity and specificity still cannot determine which kidney is affected or pinpoint the intrarenal site of injury (*8*–*11*). These limitations underscore the urgent need for an early, noninvasive, and spatially resolved method capable of detecting and localizing renal dysfunction before overt biochemical changes, especially in patients with high risks, such as those with diabetes, systemic lupus erythematosus, or those undergoing nephrotoxic drug treatments.

Imaging strategies that use renal-clearable contrast agents, from small molecules to ultrasmall nanoprobes, bridge this diagnostic blind window by providing spatially and temporally resolved intrarenal readouts (*12*, *13*). Because these agents are filtered at the glomerulus and pass through the tubules, their signal-time kinetics (from delivery to clearance) report regional function and thereby localize dysfunction, rather than yielding only whole-organ averages (*14*, *15*). Dynamic acquisitions yield quantitative, voxel-level metrics (e.g., peak enhancement, time to peak, and corticomedullary contrast) that can differentiate hemodynamic, tubular, and obstructive mechanisms within a single examination (*16*, *17*). This advantage, however, poses a clinical dilemma: Assessing kidney function requires administering a renal-clearable agent that increases osmotic load and can aggravate injury in vulnerable patients (*18*). Accordingly, translational approaches must deliver a diagnostically useful signal at reduced contrast dose, using probes that clear rapidly, bind proteins minimally, exhibit low tissue retention, and maintain predictable pharmacokinetics at low concentrations to support quantitative readouts (*19*, *20*). In short, the goal is to use as little contrast as possible while still showing when and where kidney injury happens, in a way that matches kidney physiology and minimizes extra burden on already fragile kidneys.

Renal-clearable gold nanoparticles (Au NPs) have emerged as functional reporters of kidney physiology because they combine favorable biocompatibility, tunable nano-bio interactions, rapid glomerular filtration, and minimal long-term retention (*21*, *22*). Prior studies have leveraged their intrinsic luminescence and high x-ray attenuation to estimate renal function using in vivo fluorescence (*23*–*25*) and planar x-ray imaging (*26*, *27*), respectively. However, fluorescence imaging is limited by photon scattering, depth-dependent attenuation, and autofluorescence, which restrict accurate and quantitative spatial assessment and constrain clinical translation (*28*). Planar x-ray lacks volumetric mapping and has typically required very high contrast doses (minimum dose of 1000 mg contrast/kg, ~690 mg Au/kg) to achieve adequate tracking (*27*). In contrast, computed tomography (CT) is widely used in clinical practice and provides fast, volumetric, quantitative imaging with submillimeter spatial resolution and whole-organ coverage, well suited to capturing corticomedullary heterogeneity and time-attenuation behavior (*29*). Moreover, CT technology continues to advance, with spectral/dual-energy and photon-counting systems improving sensitivity and spatial resolution and further lowering the contrast dose needed for detection (*30*). These features position renal-clearable Au NPs on CT as a practical route to early, spatially resolved mapping of kidney dysfunction under contrast-sparing conditions. However, there has not yet been a clear description of how Au NP-enhanced CT imaging patterns differ across renal dysfunctions or a simple mechanism-based interpretation that links the imaging signatures to the underlying disease process.

Here, we present a contrast-sparing CT framework that uses renal-clearable glutathione-gold nanoclusters (GSH-Au NCs) as a high-attenuation, fast-clearance probe for noninvasive, early, spatial mapping of kidney dysfunction. The physicochemical characterizations and in vivo biocompatibility of GSH-Au NCs are systematically investigated. Then, we evaluate low-dose (250 mg GSH-Au NCs/kg, 162.5 mg Au/kg) GSH-Au–enhanced CT in naïve mice and in three murine models of acute kidney injury (AKI) that represent clinically relevant pathogenic mechanisms, including local renal ischemia (prerenal), cisplatin-induced nephrotoxicity (intrarenal), and unilateral ureteral obstruction (UUO; postrenal) (*31*). For each model, we extract spatial enhancement patterns and time-attenuation curves to define early, mechanism-specific signatures. In particular, for the cisplatin nephrotoxicity model, we further test an ultralow contrast dose (50 mg GSH-Au/kg, 32.5 mg Au/kg) to minimize renal burden while preserving diagnostic performance, and we localize the dominant sites of involvement in this intrinsic model with compartment-level precision. Last, we summarize characteristic imaging features across etiologies and outline a simple, broadly applicable CT acquisition workflow suitable for early renal dysfunction assessment.

## RESULTS

### GSH-Au NCs exhibited ultrasmall size and superior x-ray attenuation ability

**Fig. 1.**
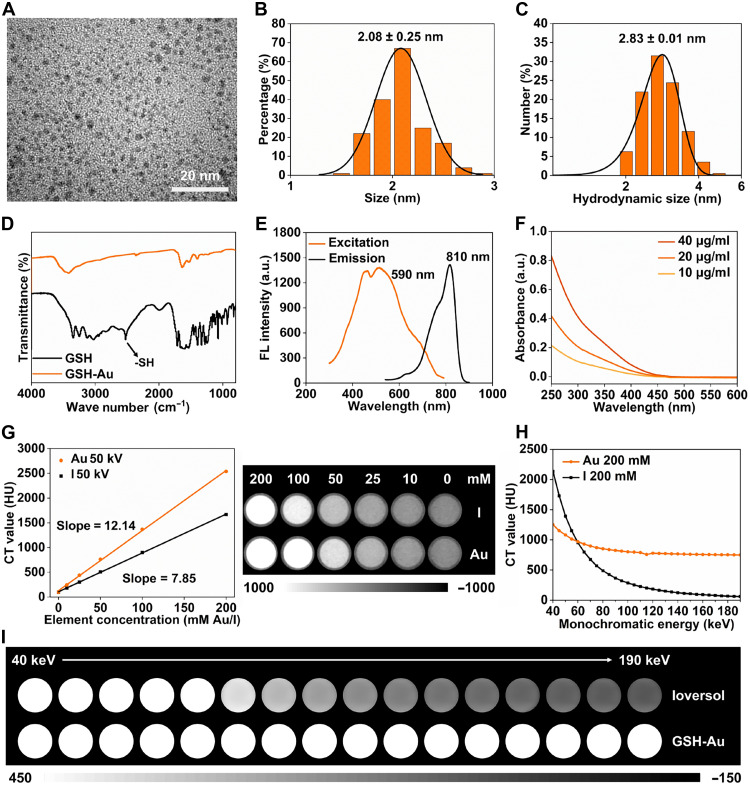
Characterization of renal-clearable GSH-Au NCs. (**A**) High-resolution transmission electron microscopy (HRTEM) image of GSH-Au NCs. (**B**) Size distribution derived from HRTEM measurements. (**C**) Hydrodynamic size distribution of GSH-Au NCs. (**D**) Fourier transform infrared (FTIR) spectroscopy of GSH-Au NCs versus free GSH. (**E**) Near-infrared (NIR) excitation-emission spectra of GSH-Au NCs. FL, fluorescence. (**F**) Ultraviolet-visible (UV-vis) absorption spectrum of GSH-Au NCs. a.u., arbitrary units. (**G**) CT phantom: attenuation-concentration plots acquired at 50 kV with corresponding phantom grayscale images. (**H**) Energy-dependent attenuation curves from monochromatic reconstructions (in kilo–electron volts) for gold and iodine references. (**I**) Corresponding monochromatic phantom images at increasing energies, matched to (H).

The ultrasmall GSH-Au NCs were synthesized by a reported protocol with minor modifications (*32*), mixing and heating glutathione (GSH) and tetrachloroauric(III) acid (HAuCl_4_) at a fixed molar ratio (details in Materials and Methods). High-resolution transmission electron microscopy showed quasispherical clusters (Fig. 1A), and size statistics gave a mean core diameter of 2.08 ± 0.25 nm (Fig. 1B). Dynamic light scattering measured a hydrodynamic diameter of 2.83 ± 0.01 nm (Fig. 1C), well below the 6-nm glomerular filtration threshold (*15*), indicating its promising renal-clearance ability. Fourier transform infrared (FTIR) spectroscopy of GSH-Au NCs compared with that of free GSH showed loss of the free-thiol band (around 2550 cm^−1^) with preserved amide features, consistent with stable covalent bond formation between Au and S (Fig. 1D). The zeta potential of GSH-Au NCs was measured to be −13.2 mV (fig. S1). Protein corona formation can substantially increase the hydrodynamic size of ultrasmall nanoparticles (*33*), thereby affecting their excretion behavior. To assess the interaction between GSH-Au NCs and serum proteins, GSH-Au NCs were incubated with bovine serum albumin (BSA) at pH 7.4 for 30 min, and binding was evaluated by agarose gel electrophoresis. As shown in fig. S2, the Coomassie Brilliant Blue-250 (CBB-250)–stained BSA bands were separated from the yellow nanocluster bands, indicating negligible binding. Due to the highly anionic glutathione ligands on their surface, GSH-Au NCs exhibited higher electrophoretic mobility than BSA. The GSH-protected Au NCs remained colloidally stable in multiple media, with no detectable clustering or precipitate formation (fig. S3). The clusters also exhibited near-infrared (NIR) photoluminescence with 590-nm excitation and 810-nm emission (Fig. 1E), and the ultraviolet-visible (UV-vis) spectrum lacked a typical plasmon peak around 520 to 530 nm, supporting a molecular-like cluster rather than larger-particle character (Fig. 1F) (*34*).

The proportion of Au in GSH-Au NCs was determined by inductively coupled plasma mass spectrometry (ICP-MS) to be 65%. As expected, the attenuation of phantoms as assessed by micro-CT increased linearly with concentration and was obviously higher for GSH-Au NCs than for the iodinated comparator Ioversol, with slopes of 12.14 Hounsfield unit (HU)/mM Au versus 7.85 HU/mM I (Fig. 1G), yielding visibly stronger contrast at matched heavy element molarities. We also tested the imaging contrast performance in a clinical CT scanner. At the routine tube voltage used in conventional spiral CT (80 to 140 kV), as the tube voltage increases, the CT value of GSH-Au NCs is significantly higher than that of Ioversol at the same element molar concentration (fig. S4). Under monochromatic reconstructions from spectral CT, GSH-Au NCs maintained higher attenuation than iodine across 60 to 190 keV with a slower decline in CT values at higher energies (Fig. 1H), corroborated by the corresponding phantom images at increasing energies (Fig. 1I). Collectively, these results show that the modified synthesis yields ultrasmall GSH-Au NCs with stable surface chemistry and dose-efficient CT contrast, supporting the application for noninvasive, contrast-sparing imaging of kidney diseases.

### Renal-clearable GSH-Au NCs generate robust intrarenal CT contrast

**Fig. 2.**
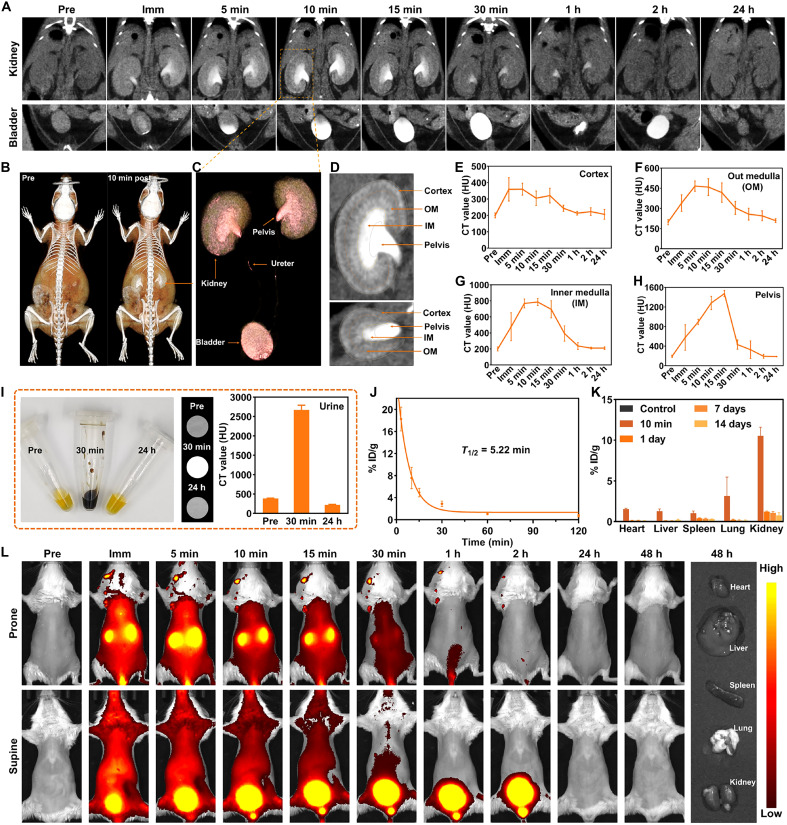
In vivo imaging tracking of renal-clearable GSH-Au NCs. (**A**) Serial CT images of the kidneys and bladder before and at different time points after intravenous dosing (325 mg Au/kg). Imm, immediately. (**B**) Whole-body volume rendered images before and 10 min after injection. (**C**) Pseudocolor volume rendering (VR) of the isolated urinary systems (kidneys, renal pelvis, ureters, and bladder). (**D**) Renal segmentation based on the GSH-Au–enhanced CT image: cortex, outer medulla (OM), inner medulla (IM), and pelvis. (**E** to **H**) Time-attenuation curves for cortex (E), OM (F), IM (G), and pelvis (H). *n* = 3 per time point. (**I**) Urine samples were collected at pre-, 30 min, and 24 hours postinjection with corresponding CT images and HU measurements. *n* = 3 per time point. (**J**) Blood pharmacokinetics showing rapid clearance with an apparent blood half-life *T*_1/2_ = 5.22 min. *n* = 3 per time point. (**K**) Biodistribution of Au at 10 min, 1 day, 7 days, and 14 days postinjection and in control, noninjected animals. *n* = 3 per time point. (**L**) Fluorescence imaging (prone and supine) from pre to 48 hours postinjection, with ex vivo organ fluorescence at 48 hours. h, hours.

We first benchmarked against clinically used iodinated contrast dosing (e.g., Ioversol: 636 to 741 mg/kg, 300 to 350 mg I/kg) by imaging naïve mice after administering GSH-Au NCs at 500 mg/kg (~325 mg Au/kg). Following intravenous dosing, multi–time-point dynamic CT showed immediate cortical enhancement with progressive corticomedullary filling. The urinary bladder became obviously hyperattenuating at 5 min postinjection and remained enhanced through 2 hours (Fig. 2A). Owing to the strong contrast of GSH-Au NCs and the high spatial resolution of CT, the kidney parenchyma, renal pelvis, ureter, and bladder were delineated (Fig. 2, B and C), facilitating structural visualization of the urinary tract. The intrarenal distribution of GSH-Au NCs further enabled robust compartmentalization into cortex, outer medulla (OM), inner medulla (IM), and pelvis (Fig. 2D), spatial resolution scarcely achievable with prior fluorescence or planar x-ray imaging (*25*, *26*). The dynamic CT-contrast curves derived from these compartments revealed a rapid wash-in of the contrast agent followed by washout, with distinct time-to-peak values: The cortex peaked immediately after injection (Fig. 2E), the OM at about 5 min (Fig. 2F), the IM at about 10 min (Fig. 2G), and the pelvis at about 15 min (Fig. 2H), consistent with the expected filtration-to-collection transit along the nephron. The sharp compartment boundaries and staggered kinetics provide a basis for localizing parenchymal injury. The probe exhibited rapid elimination from the blood (half-life *T*_1/2_ = 5.22 min) via the kidneys, as evidenced by high accumulation in the urinary bladder (Fig. 2, I and J, and fig. S5). Quantification of urinary Au confirmed brisk excretion, with a 2-hour renal clearance of 59.1%. Only transient enhancement was observed in the liver, spleen, lung, and heart, most likely due to intravascular perfusion, demonstrating the low nonspecific distribution of GSH-Au NCs (fig. S6). Ex vivo biodistribution (Fig. 2K and fig. S7) emphasized a kidney-dominant, transient exposure: percentage of injected dose (% ID)/organ in kidney was maximal at 10 min, fell sharply by 1 day (less than 0.4%), and returned to near-background by 7 to 14 days, which is a key safety advantage over many conventional nanoparticles (*35*).

Whole-body fluorescence imaging provided consistent dynamics, with bilateral renal signal evident within 30 min and negligible residual whole-body signal by 24 to 48 hours (Fig. 2L). However, compared with CT, fluorescence was limited to rough renal outlines and could not resolve corticomedullary layering. Because of shallow tissue penetration and scattering, organs in nondepilated regions were essentially invisible, and we often had to reposition animals to separately visualize the kidneys and bladder. These nontomographic, surface-weighted constraints markedly limit quantitative assessment and hinder clinical translation compared with CT.

Collectively, GSH-Au NCs generate robust intrarenal contrast with compartment-specific enhancement that resolves corticomedullary and pelvic kinetics in vivo. Pharmacokinetic and biodistribution analyses show rapid urinary clearance, minimal off-target exposure, and no sustained tissue retention. Together, these features enable early, spatially resolved mapping of renal dysfunction while reducing the risk of deposition-related adverse effects.

### Contrast-sparing CT precisely maps the injury territory of local renal ischemia

**Fig. 3.**
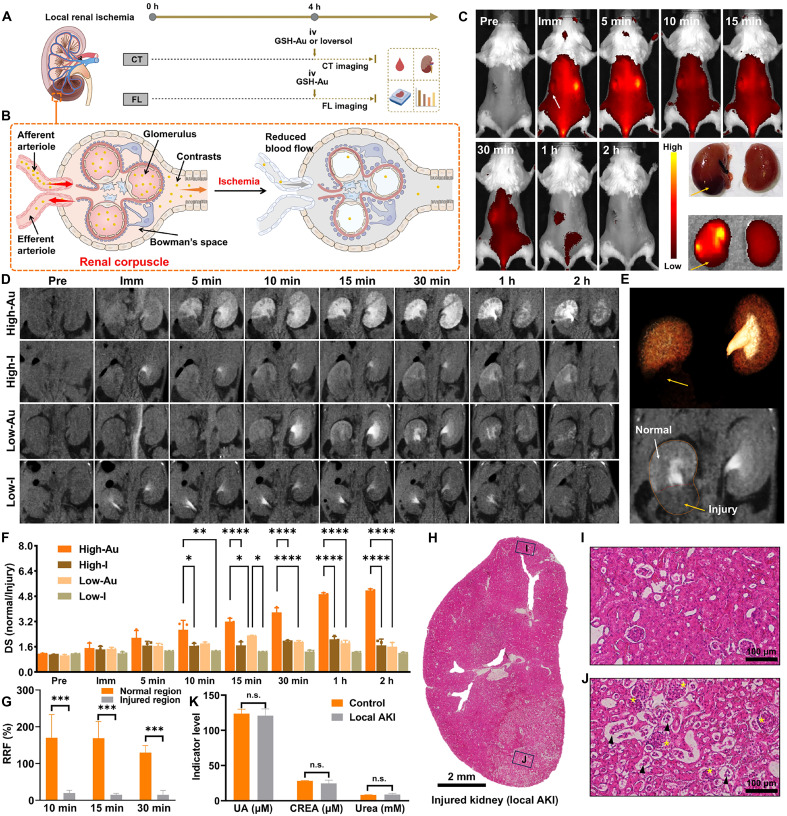
Mapping the injury territory of local renal ischemia with GSH-Au NCs. (**A**) Workflow for the prerenal AKI model: segmental renal artery ligated for 4 hours, followed by intravenous (iv) dosing of GSH-Au NCs or Ioversol, serial imaging, hematologic, and histologic analysis. FL, fluorescence. (**B**) Schematic of contrast delivery and glomerular filtration within the renal corpuscle under normal flow versus ischemia. (**C**) Fluorescence whole-body imaging at predefined time points. The white arrow indicates the injured kidney, and the yellow arrows indicate the locally injured region. (**D**) Serial contrast-enhanced CT images acquired with four dosing regimens: High-Au (325 mg Au/kg), High-I (Ioversol at a molar-equivalent iodine dose to 325 mg Au/kg, 209 mg I/kg), Low-Au (162.5 mg Au/kg), and Low-I (Ioversol at a molar-equivalent iodine dose to 162.5 mg Au/kg, 104.5 mg I/kg). (**E**) VR and a representative coronal CT slice after Low-Au administration. The dashed line outlines the boundary between normal and injured parenchyma. The yellow arrows indicate the locally injured region. (**F**) Quantification based on a normal-to-injury HU ratio within the selected region of interest (ROI). *n* = 3 per time point. *P < 0.05; **P < 0.01; ****P < 0.0001. (**G**) Quantification of RRF in the affected kidney at serial time points after Low-Au administration. *n* = 3 per time point. ***P < 0.001. (**H** to **J**) Hematoxylin and eosin (H&E) histology of the locally injured region and the normal region. Black arrowheads mark necrotic, sloughed tubular epithelial cells, and yellow asterisks denote collapsed/poorly patent glomerular capillary loops. (**K**) Postimaging serum biochemistry [creatinine (CREA) and uric acid (UA)]. *n* = 3 per group; n.s., not significant. Cartoon images in (A) and (B) were created in BioRender. S. Sun (2026), https://BioRender.com/l7bom6p. h, hours.

We induced early, local renal ischemia by ligating the segmental renal artery branch for 4 hours (Fig. 3A). Mice then received intravenous dosing of GSH-Au NCs, followed by serial contrast-enhanced CT and fluorescence imaging. An iodine comparator (Ioversol) was administered at an Au-molarity–matched dose and imaged with the same CT protocol. Imaging was followed by hematologic assays and histology. Mechanistically, contrast reaches nephrons via afferent arterioles and generates a CT signal from both vascular perfusion and tubular luminal content after glomerular filtration (Fig. 3B) (*15*). Segmental arterial ligation acutely depresses preglomerular inflow and glomerular capillary pressure in the affected territory, sharply reducing both tissue perfusion and filtered tubular delivery.

As shown in Fig. 3C, fluorescence imaging captured the expected kinetic asymmetry but lacked spatial specificity. After injecting GSH-Au NCs (325 mg Au/kg), the injured kidney showed lower fluorescence at 5 min and delayed washout, yielding higher residual signal at ~1 hour versus the contralateral side. Nevertheless, in vivo fluorescence imaging delineated only a coarse renal outline, and the injury locus was essentially indiscernible on live imaging and could be identified only ex vivo, where the lower pole of the affected kidney appeared hypoenhanced, underscoring the limited utility of fluorescence for clinical renal mapping.

By contrast, CT at the same 325 mg Au/kg dose provided high-fidelity lesion mapping. The injured kidney exhibited heterogeneous enhancement with normally enhancing mid/upper poles and a weakly enhancing lower pole, forming a sharply demarcated perfusion defect that persisted from about 5 min to 2 hours (Fig. 3D). With equimolar iodine, lesion-to-normal contrast was smaller and boundary definition poorer, consistent with iodine’s lower x-ray attenuation per mole. To minimize the excretion burden on the injured kidney, we further evaluated the potential of low-contrast-dose CT for assessing local injury. Notably, low-dose GSH-Au NCs (162.5 mg Au/kg) still delineated the ischemic territory and corticomedullary architecture. Low-dose GSH-Au NCs were comparable to high-dose iodine and substantially superior to low-dose iodine (Fig. 3D). Volume rendering (VR) further highlighted the signal void at the lower pole with preserved enhancement elsewhere, consistent with selective branch ligation (Fig. 3E).

For quantitative comparison, we extracted time-attenuation curves from normal and injured renal parenchyma in mice given different contrast agents (fig. S8) and defined a difference score (DS) as the ratio of the CT value in normal to injured parenchyma within the same kidney (higher DS indicates greater separation). As summarized in Fig. 3F, high-dose GSH-Au (325 mg Au/kg) yielded the highest DS throughout the 10-min to 2-hour imaging window, low-dose GSH-Au (162.5 mg Au/kg) achieved DS values comparable to high-dose iodine, and, after 15 min, its DS exceeded that of low-dose iodine. To further quantify the degree of renal damage, we defined a residual renal function (RRF) in low-dose GSH-Au–treated mice as the CT attenuation of a region of interest (ROI) expressed as a percentage of the contralateral parenchyma at a matched location (lower RRF indicates worse kidney function). As shown in Fig. 3G, RRF in injured regions of the affected kidney was significantly lower than that of normal regions within the same kidney at 10, 15, and 30 min after injection. Notably, RRF in the normal-appearing regions of the affected kidney exceeded 100%, suggesting potential compensatory function.

Pathology confirmed the imaging readout: Hematoxylin and eosin (H&E) sections from the affected kidney region showed collapsed/poorly patent glomerular capillary loops and tubular epithelial necrosis and sloughing (Fig. 3, H to J). Despite these focal changes, serum creatinine (CREA), urea, and uric acid (UA) remained unchanged in the early window (Fig. 3K), underscoring biomarker lag and the high sensitivity and spatial resolution of CT with low-dose GSH-Au NCs for mapping and quantifying local prerenal AKI. Clinically, delineating the precise extent and quantifying the degree of focal ischemia enable early risk stratification and guide targeted, nephron-sparing management and monitoring before global kidney function declines.

### Ultralow-dose GSH-Au NCs enable the early mapping of intrarenal AKI

**Fig. 4.**
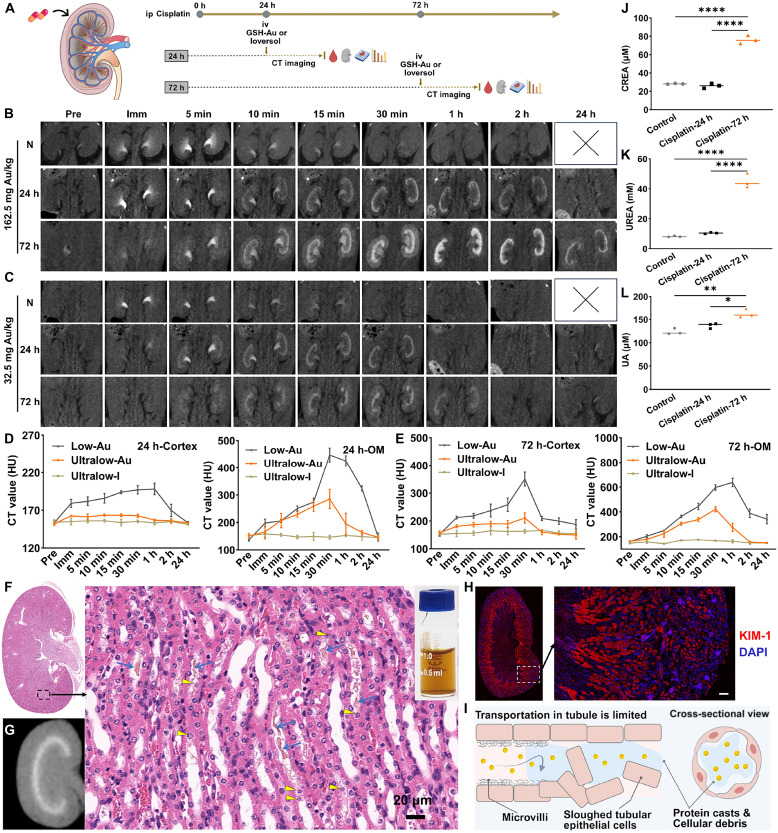
Compartment-level mapping of drug-induced intrarenal AKI with ultraslow-dose GSH-Au–enhanced CT. (**A**) Workflow for the intrinsic (drug-induced) model: cisplatin administered intraperitoneally (ip), followed by intravenous (iv) injection of GSH-Au NCs or Ioversol, serial CT imaging, and subsequent hematologic and histologic analyses. (**B** and **C**) Serial contrast-enhanced CT acquired with low dose (162.5 mg Au/kg) and ultralow dose (32.5 mg Au/kg) GSH-Au NCs; for each dose, scans were performed in naïve mice (N) and in cisplatin-treated mice at 24 and 72 hours postcisplatin. (**D** and **E**) Compartment-based time-attenuation curves for cortex and OM after contrast injection in cisplatin-treated mice at 24 hours (D) and 72 hours (E). *n* = 3 per time point. (**F**) H&E staining of the kidney in 24-hour cisplatin-treated mice postinjection of ultralow-dose GSH-Au NCs (32.5 mg Au/kg). Blue arrows indicate composites of GSH-Au NCs with proteins/debris, and yellow arrowheads indicate collapsed/sloughed tubular epithelial cells. The inserted picture is a photograph of a yellow-brown GSH-Au NCs aqueous solution. (**G**) Corresponding ex vivo CT image of the kidney in (F). (**H**) KIM-1 immunofluorescence of the kidney in 24-hour cisplatin-treated mice postinjection of ultralow-dose GSH-Au NCs (32.5 mg Au/kg). Scale bar, 50 μm. DAPI, 4′,6-diamidino-2-phenylindole. (**I**) Schematic illustrating the potential mechanism of delayed tubular clearance of GSH-Au NCs during drug-induced AKI. (**J** to **L**) Postimaging serum biochemistry (creatinine, urea, and uric acid). *n* = 3 per group. *P < 0.05; **P < 0.01; ****P < 0.0001. Cartoon images in (A) and (I) were created in BioRender. S. Sun (2026), https://BioRender.com/c2ohp87.

As outlined in the schematic (Fig. 4A), mice received intraperitoneal cisplatin and underwent CT at 24 and 72 hours with dynamic acquisitions after intravenous injection of GSH-Au NCs or Ioversol. Because drug nephrotoxicity can be bilateral, we started with a contrast-sparing dose of 162.5 mg Au/kg GSH-Au NCs, which had already shown good performance in the prerenal AKI model. In naïve mice, this dose produced the expected rapid, mild enhancement in kidney parenchyma, which was quickly excreted by the kidneys (Fig. 4B). By 1 to 2 hours postinjection, renal parenchymal signals had largely returned to baseline levels.

In cisplatin-treated mice at 24 hours, injection of the same dose of GSH-Au NCs revealed a band-like hyperattenuation in the OM starting at 10 min, becoming more pronounced through 2 hours and disappearing by 24 hours (Fig. 4, B and D). This feature was not observed in naïve mice. For cisplatin-treated mice at 72 hours, CT scans showed that enhancement in cortex and OM increased within the first 30 min, with the OM significantly brighter than the cortex (Fig. 4, B and E). By 1 hour, cortical signals declined, whereas outer-medullary enhancement intensified and persisted. Even at 24 hours postinjection, a distinct band of enhancement remained in the OM, indicating sustained retention in this region.

Given that cisplatin compromises both kidneys, further reducing contrast burden is clinically meaningful. We therefore evaluated an ultralow dose of 32.5 mg Au/kg GSH-Au NCs (Fig. 4, C to E). In cisplatin-treated mice at 24 hours, the OM was visible on 5- to 30-min frames. At the 72-hour model, the same region showed delayed peak enhancement and prolonged retention compared with the cortex and pelvis. However, by 2 hours postinjection, the hyperattenuating band in the OM was no longer distinguishable, suggesting that most of the contrast agent had already been cleared from the kidneys. Notably, naïve mice imaged at 32.5 mg Au/kg did not exhibit a band-like OM pattern, confirming the injury specificity of the observed changes. When compared side by side, a dose of 32.5 mg Au/kg (Ultralow-Au) showed less conspicuity than 162.5 mg Au/kg GSH-Au (Low-Au) but was still superior to equimolar iodine (Ultralow-I, 20.9 mg I/kg; fig. S9), confirming that diagnostic mapping can be effectively maintained at a significantly lower Au dose.

These results demonstrated that, in the drug-induced nephropathy model, both high and low doses of GSH-Au NCs exhibited significant accumulation or delayed clearance in the OM, strongly indicating that this region may be a critical site for drug-induced injury. Correlative histology further confirmed this finding: H&E staining identified outer-medullary tubular injury (proteinaceous casts and sloughed tubular endothelial debris) (Fig. 4F), colocalizing with ex vivo kidney CT hyperenhancement (Fig. 4G). KIM-1 is a highly sensitive marker of tubular injury (*36*), and immunofluorescence showed robust KIM-1 expression in renal tubules, especially in the OM area (Fig. 4H). The distinct yellow-brown color of GSH-Au NCs, along with their obvious retention in the OM, facilitated easy identification of Au NCs in H&E-stained kidney sections (Fig. 4F). The GSH-Au NCs predominantly localized in renal tubules within the OM, which are likely aggregated together with protein casts or cellular debris (Fig. 4I) (*27*). Moreover, the renal tubule’s role in water reabsorption likely concentrates the GSH-Au NCs in the affected regions, enabling significantly high-density CT mapping of the tubular injury area. This phenomenon facilitates the early spatial visualization of drug-induced renal damage.

The biology readouts supported the early imaging sensitivity of both GSH-Au NCs doses (32.5 and 162.5 mg Au/kg) (Fig. 4, K and L). In cisplatin-treated mice at 24 hours, serum creatinine and urea remained near baseline while CT already depicted outer-medullary changes (Fig. 4, B and C). By 72 hours, both biomarkers increased as CT showed progression and persistence of the outer-medullary abnormality.

To further evaluate the clinical translatability across clinically relevant conditions, we performed in vivo imaging on a clinical CT scanner using the same ultralow dose of GSH-Au NCs (32.5 mg Au/kg) in the early-stage cisplatin-AKI model (24 hours). Scans acquired at 80, 100, and 120 kV with a clinically relevant reconstruction (0.5-mm slice thickness) consistently revealed preferential accumulation of GSH-Au NCs in the OM, producing a characteristic ring-like enhancement pattern (fig. S10, A and B). Quantitatively, the outer-medullary contrast-to-noise ratio (CNR) increased markedly after injection, peaked at ~30 min, and declined toward baseline by 2 hours, consistent with rapid renal transit and excretion (fig. S10C). These clinical-CT results demonstrate that the proposed contrast-sparing strategy remains robust under clinical scanning conditions, supporting its translational potential for early detection and spatial mapping of drug-induced tubular injury while minimizing contrast burden in at-risk patients.

Collectively, starting at 162.5 mg Au/kg and stepping down to 32.5 mg Au/kg preserve early, compartment-level detection of drug-induced intrarenal AKI while further minimizing contrast dose, a clinically relevant advantage for patients with bilateral or evolving nephrotoxicity who require serial monitoring.

### Low-dose GSH-Au NC–enhanced CT for early spatial mapping of postrenal AKI

**Fig. 5.**
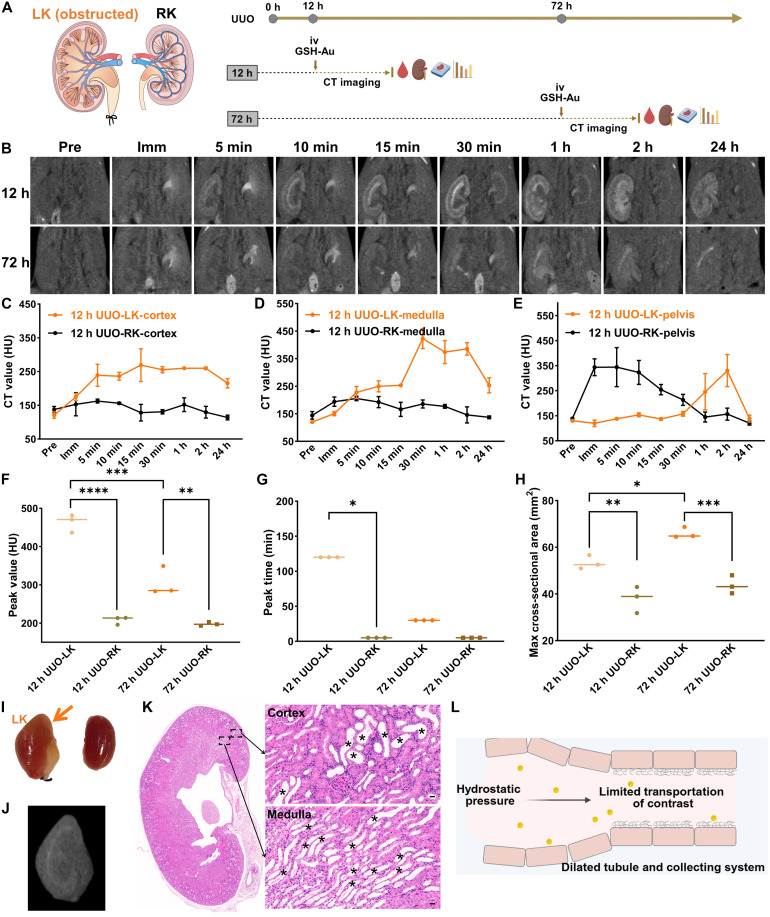
Mapping morphologic and signal changes of postrenal AKI with low-dose GSH-Au–enhanced CT. (**A**) Workflow for the UUO (postrenal AKI) model: unilateral ureter ligation for 12 or 72 hours, followed by intravenous (iv) GSH-Au NCs (162.5 mg Au/kg), serial CT imaging, and subsequent hematologic and histologic analyses. (**B**) Serial contrast-enhanced CT acquired at predefined time points in mice 12 and 72 hours post-UUO. (**C** to **E**) Compartment-based time-attenuation curves (cortex, medulla, and pelvis) after contrast injection in 12-hour UUO mice. LK, left kidney (obstructed); RK, right kidney (nonobstructed). *n* = 3 per time point. (**F** to **H**) Summary metrics of both kidneys after GSH-Au NCs: peak enhancement (F), time to peak (G), and max renal cross-sectional area (H). *n* = 3 per group. *P < 0.05; **P < 0.01; ***P < 0.001; ****P < 0.0001. Ex vivo photograph (**I**) and CT image (**J**) of the obstructed kidney at 30 min after low-dose GSH-Au NCs (162.5 mg Au/kg) administration at 72-hour UUO model. The orange arrow points to the obstructed kidney. (**K**) H&E staining of the obstructed kidney at 72-hour UUO model. Black asterisks indicate dilated renal tubules. Scale bar, 20 μm. (**L**) Schematic illustrating the potential mechanism underlying CT imaging patterns in postrenal AKI. Cartoon images in (A) and (L) were created in BioRender. S. Sun (2026), https://BioRender.com/ubg6hmc. h, hours.

We established a UUO mouse model by ligating one side of the ureter for 12 or 72 hours and then performed serial contrast-enhanced CT at predefined time points after intravenous GSH-Au NCs (162.5 mg Au/kg) (Fig. 5A). At 12 hours post-UUO, CT showed marked asymmetry. The contralateral (nonobstructed) kidney demonstrated prompt visualization of the renal pelvis, a transient parenchymal enhancement, and returned to near baseline by 24 hours. In contrast, the obstructed kidney exhibited delayed enhancement of the cortex and medulla. Within 2 hours, the medullary signal exceeded the cortical signal, and parenchymal hyperattenuation persisted at 24 hours. The ipsilateral pelvis enhanced late (becoming obvious at 1 to 2 hours) (Fig. 5, B to E). When obstruction was prolonged to 72 hours, the obstructed kidney retained the delayed enhancement, although the peak intensity was lower than that at 12 hours (fig. S11). Morphologically, the obstructed kidney was enlarged and the renal pelvis more dilated, with both changes more pronounced at 72 hours.

To further quantify these differences, we extracted peak enhancement and time to peak from time-attenuation curves of parenchyma (cortex and medulla) and measured the maximum renal cross-sectional area. The 12-hour UUO obstructed kidney showed higher peak enhancement (463 HU versus 208 HU) and longer time to peak (2 hours versus 5 min) than the contralateral kidney (Fig. 5, F and G). The 72-hour UUO obstructed kidney showed the same directionality but lower peak CT values (306 HU) than the 12-hour group (463 HU), consistent with more severe obstruction limiting parenchymal delivery. In both models, the ipsilateral maximum cross-sectional area was significantly greater than the contralateral side and increased with obstruction duration (Fig. 5H).

Ex vivo assessment corroborated imaging. The obstructed kidney was visibly enlarged relative to the contralateral kidney (Fig. 5, I and J). H&E demonstrated obvious tubular dilatation in both cortex and medulla (Fig. 5K and fig. S12). It can be inferred that urinary stasis and elevated intratubular hydrostatic pressure slow the intrarenal clearance of GSH-Au NCs (Fig. 5L), producing the observed triad of pelvic dilatation, renal enlargement, and delayed parenchymal enhancement. Notably, single morphologic changes do not always indicate renal injury. For example, dilation of the renal pelvis seen on imaging may reflect an anatomic variant (extrarenal pelvis) rather than pathology (*37*). In contrast, GSH-Au–enhanced CT provides both morphologic information and quantitative changes in renal parenchymal attenuation (CT values), enabling robust discrimination between anatomic variants and true renal injury, thereby reducing the risk of misdiagnosis.

Despite these clear imaging and histopathologic alterations, serum creatinine, urea, and uric acid remained unchanged at both 12 and 72 hours post-UUO (fig. S13), likely due to compensation by the contralateral kidney. Together, these findings indicated that low-dose GSH-Au–enhanced CT sensitively detects early (12 hours), spatially resolved dysfunction in postrenal AKI, supporting its use for subclinical diagnosis and longitudinal monitoring of obstructive nephropathy.

### GSH-Au NCs showed good biocompatibility in vivo

**Fig. 6.**
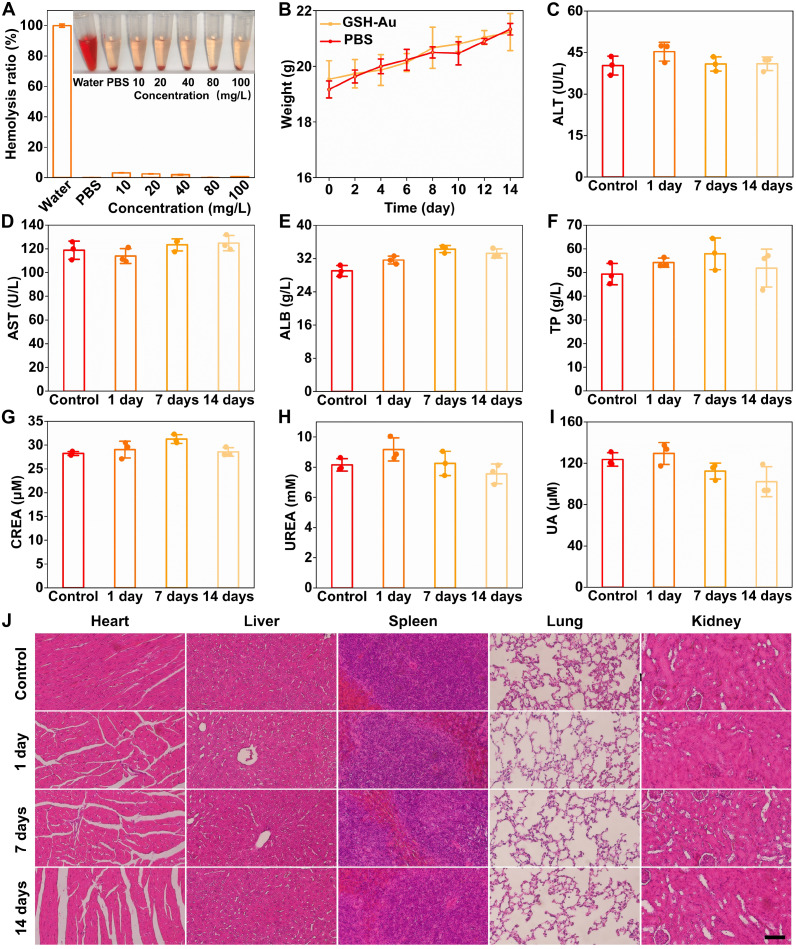
In vivo biocompatibility of GSH-Au NCs. (**A**) Red blood cell hemolysis after incubation with GSH-Au NCs (10 to 100 mg/liter); water and PBS served as positive and negative controls, respectively. *n* = 3 per group. (**B**) Body weight over 14 days in GSH-Au–treated and PBS control mice. *n* = 3 per group. (**C** to **I**) Serum biochemistry at baseline (control), 1 day, 7 days, and 14 days postinjection: alanine aminotransferase (ALT), aspartate aminotransferase (AST), albumin (ALB), total protein (TP), serum creatinine (CREA), urea (UREA), and uric acid (UA). *n* = 3 per group. (**J**) Representative H&E sections of the heart, liver, spleen, lung, and kidney at the same time points (scale bars, representative). L, liter.

Although Au NCs are widely regarded as biocompatible owing to their intrinsic inertness, the relatively high mass doses used for CT imaging warrant systematic safety evaluation at the upper dosing range. We assessed the in vivo biocompatibility of GSH-Au NCs (325 mg Au/kg) by profiling hemocompatibility, longitudinal serum biochemistry, and multiorgan histology. As shown in Fig. 6A, GSH-Au NCs exhibited good hemocompatibility across 10 to 100 mg/liter. The hemolysis ratio remained at background levels comparable to phosphate-buffered saline (PBS) and far below the 5% nonhemolytic threshold. The mice treated with GSH-Au NCs maintained normal growth trajectories with body weights increasing similarly to PBS controls over 14 days (Fig. 6B). Serum biochemistry revealed no evidence of hepatic or renal toxicity following a 325 mg Au/kg intravenous dose. Hepatocellular injury markers (ALT and AST) remained within physiological ranges at 1, 7, and 14 days (Fig. 6, C and D), while synthetic function indices (ALB and TP) were unchanged (Fig. 6, E and F). Renal function parameters (CREA, UREA, and UA) likewise showed no meaningful deviation from baseline or PBS controls throughout follow-up (Fig. 6, G to I). Histopathology demonstrated the benign profile. H&E staining demonstrated preserved myocardial fibers without necrosis or inflammatory infiltrates, intact hepatic lobular architecture without hepatocyte ballooning or periportal inflammation, normal splenic white/red pulp organization, aerated lungs without alveolar wall thickening or hemorrhage, and kidneys with intact glomeruli and tubules lacking necrosis, cast formation, or interstitial infiltration at all time points (Fig. 6J).

Together, these data indicate that GSH-Au NCs are nonhemolytic and well tolerated in vivo and do not cause detectable hepatic or renal toxicity or off-target tissue injury up to 14 days after a single 325 mg Au/kg dose, which is consistent with their rapid urinary clearance and minimal long-term retention demonstrated in biodistribution studies. This safety profile supports their use as CT probes for spatial mapping of renal dysfunction.

## DISCUSSION

Early, noninvasive, and spatially resolved visualization of renal dysfunction is clinically pivotal (*2*). By showing where injury occurs and how quickly it evolves, it supports etiologic attribution, guides drug selection or cessation, and informs the need and timing of interventions (*7*). Here, we demonstrate a contrast-sparing CT approach in which renal-clearable GSH-Au NCs generate compartment-level maps of kidney dysfunction, localizing injury and inferring etiology before serum biomarkers change.

Current tools for assessing kidney function are complementary but leave important gaps. Routine lab indicators, such as serum creatinine, blood urea nitrogen, uric acid, and creatinine-based eGFR, are inexpensive and ubiquitous, yet they lag injury, assume near-steady state, and report whole-kidney averages (*3*, *4*). The emerging tubular or glomerular biomarkers (e.g., cystatin C, urinary albumin-to-creatinine ratio, β2-microglobulin, *N*-acetyl-β-d-glucosaminidase, KIM-1, and neutrophil gelatinase–associated lipocalin) significantly improve the sensitivity and risk stratification (*8*, *38*) but still cannot exactly tell which kidney or which area is affected. Medical imaging can help to localize the injury. The convenient and cost-effective ultrasound quickly delineates renal anatomy (e.g., dilation of the ureter/pelvis in the case of obstruction), while the Doppler resistance index reflects regional perfusion (*39*). However, the diagnosis accuracy of ultrasound is operator-dependent and limited in obese patients or when bowel gas overlies the kidneys (*40*). Magnetic resonance imaging (MRI) with diverse scanning sequences can sensitively show segmental abnormalities without radiation, yet access, scan time, motion sensitivity, and cost limit the routine and urgent use (*41*). Nuclear imaging, like planar renography, single-photon emission computed tomography, and positron emission tomography, provides functional curves and splits renal function, but spatial resolution is low for intrarenal mapping (*42*). Combining laboratory indicators with imaging is a practical strategy for assessing renal dysfunction. Our aim is not to replace these tools but to complete the diagnostic ladder with a fast, quantitative, spatial map when localization matters. CT is rapid and widely available, provides volumetric, quantitative readouts (in HU) with submillimeter resolution and whole-organ coverage, and yields standardized, less operator-dependent images. Accordingly, the proposed contrast-sparing CT with renal-clearable GSH-Au NCs combines the high sensitivity of laboratory tests with the spatial precision of imaging. On the one hand, it can screen early renal injury when laboratory indices are normal or equivocal. On the other hand, it serves as a valuable adjunct to abnormal lab indicators, confirming the injury, clarifying the injury extent (bilateral, unilateral, or compartment-level regions), and inferring the likely mechanism of dysfunction, even in questionable cases.

Despite the abovementioned advantages, the value of contrast-enhanced CT in compromised kidneys is to some extent limited by contrast burden (*43*). Against this backdrop, a practical CT contrast for renal imaging must meet at least three requirements simultaneously: high x-ray attenuation to preserve lesion-background separation at reduced dose, reliable renal clearance to avoid prolonged tissue retention, and predictable low-dose pharmacokinetics to support quantification (*44*–*46*). Several renal-clearable, high-*Z* CT/x-ray agents have been reported, including formulations based on bismuth (Bi) (*47*–*52*), tantalum oxide (TaOx) (*53*), hafnium (Hf) (*54*), silver (Ag) (*55*), molybdenum (Mo) (*56*), and gold (Au) (*57*), alongside iodinated small molecules (*58*). Each class trades off elemental loading, particle size, and clearance. Hf-, Ag-, and Ta-based nanodots can deliver strong attenuation but often push the size-dose envelope to maintain visibility while ensuring glomerular filtration (*53*–*55*); some platforms show more reticuloendothelial system uptake or slower elimination (*59*, *60*). Iodine is familiar and accessible but loses conspicuity as the dose is cut. By contrast, our GSH-Au NCs satisfy all three criteria. First, as our findings demonstrated, the x-ray attenuation of Au is substantially higher than that of iodine at the same molar concentration, evidenced by greater CT imaging sensitivity. In addition, the effective Au mass fraction of the clusters was as high as 65%, further enabling dose efficiency. Second, the hydrodynamic diameter was 2.8 nm, well below the 6- to 8-nm filtration threshold (*15*), promoting rapid glomerular filtration. As expected, after intravenous dosing, robust pelvis and bladder enhancement appeared within minutes, and biodistribution showed minimal extra-renal accumulation out to 2 weeks. Third, the near-linear HU-concentration relationship in phantoms translated in vivo into compartment-specific time-attenuation curves (cortex, OM, IM, and pelvis) that captured perfusion, filtration, tubular transit, and outflow within a single exam. In addition, the intrinsic inertness of the Au element confers good biosafety, as supported by our in vivo biocompatibility assessments. Collectively, these results demonstrate that GSH-Au–enhanced CT provides a dose-efficient and biocompatible route to structural and functional mapping of kidneys that meets the physiological and practical constraints of clinical conditions.

**Fig. 7.**
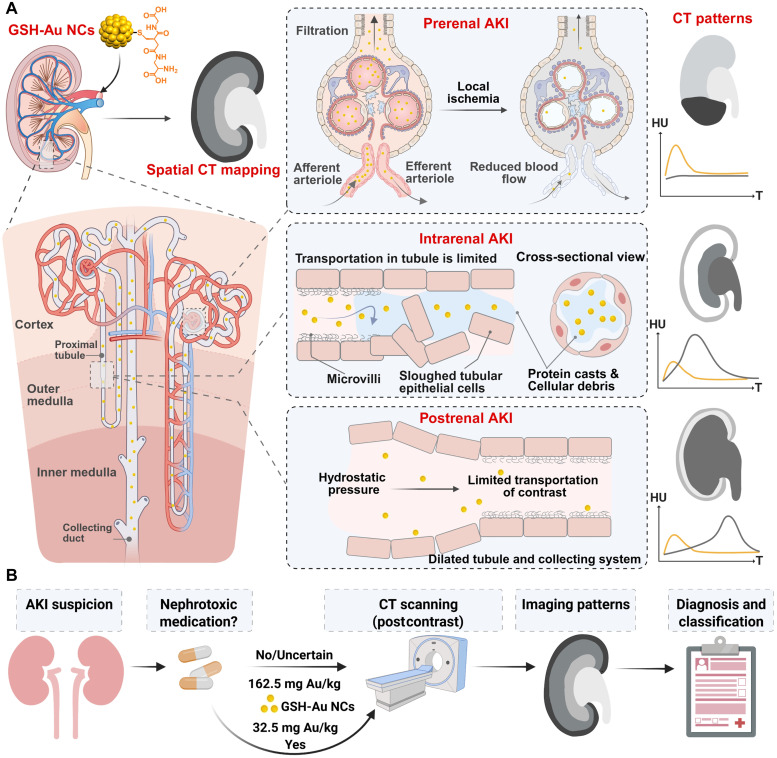
Contrast-sparing CT with renal-clearable GSH-Au NCs for early spatial mapping of renal dysfunction. (**A**) Schematic illustration of intrarenal distribution/clearance of GSH-Au NCs in the normal kidney and different AKI kidneys: prerenal (ischemic), intrinsic (tubular), and postrenal (obstructive). These models have distinguishable CT morphologic signatures and representative compartment-based time-attenuation profiles. In time-attenuation curves of the prerenal AKI model: Yellow line indicates the normal renal parenchyma; gray line indicates the injured renal parenchyma. In time-attenuation curves of intrarenal AKI model: Yellow line indicates the OM in healthy kidney; gray line indicates the OM in the injured kidney. In time-attenuation curves of postrenal AKI model: Yellow line indicates the renal parenchyma in healthy kidney; gray line indicates the renal parenchyma in obstructed kidney. (**B**) Streamlined and general low-dose GSH-Au-CT workflow for AKI screening and classification. Cartoon images in (A) and (B) were created in BioRender. S. Sun (2026), https://BioRender.com/t090r8m.

The clinical translation of nanosized contrast agents is a major challenge (*61*). In our study, at least two essential findings support the potential clinical translation of GSH-Au–enhanced CT. The first one is the dose efficiency. Starting from a conservative 162.5 mg Au/kg (0.825 mmol Au/kg) that performed well across all three AKI models, we stepped down to 32.5 mg Au/kg (0.165 mmol Au/kg) in drug-induced AKI without loss of diagnostic separability, yet equimolar iodine loses the diagnostic ability. Notably, 32.5 mg Au/kg is an ultralow CT dose: It is well below typical iodinated contrast dosing (300 to 350 mg I/kg = 2.36 to 2.76 mmol I/kg) (*62*), below the GSH-Au NCs dose commonly used for renal fluorescence imaging (100 mg Au/kg) (*25*), just about 1/20th of that needed for planar x-ray renal imaging (690 mg Au/kg) (*26*, *27*), and also below the dose for CT imaging in other reports (*27*, *63*). On a molar basis, 0.165 mmol Au/kg (32.5 mg Au/kg) is even comparable to clinical gadolinium (Gd)–based contrast ranges for MRI (0.1 to 0.2 mmol Gd/kg) (*64*). This step-down strategy addresses a long-standing problem in renal functional imaging: The very organs that we need to assess are the least able to tolerate contrast load. Lower dose improves the feasibility of serial exams, reduces the chance of prolonged intrarenal retention, and aligns with the realities of patients with bilateral or evolving nephrotoxicity who require longitudinal monitoring. Using the same ultralow dose (32.5 mg Au/kg), we further confirmed on a clinical CT scanner (80 to 120 kV, 0.5-mm slice thickness) that the characteristic outer-medullary enhancement in early cisplatin-AKI remains detectable, supporting clinical translatability. Second, mechanism-specific imaging patterns enable practical AKI classification rather than guesswork. With GSH-Au–enhanced CT, each etiology produced a reproducible morphologic signature and a distinct time-attenuation profile (Fig. 7A). In prerenal ischemia, segmental inflow falls abruptly, lowering glomerular capillary pressure and starving downstream tissue of contrast (*65*). The result is a sharply demarcated hypoenhancement that respects segmental anatomy. In drug-induced intrinsic AKI, tubular necrosis and epithelial sloughing with abundant protein casts and debris slow intratubular transit (*27*). Coupled with water reabsorption, the concentrated GSH-Au in tubules, most prominently in the OM, yields a band-like outer-medullary pattern with blunted peaks and delayed washout. This also explains why drug-induced renal injury remains obvious on CT imaging even at an ultralow dose of GSH-Au NCs (32.5 mg Au/kg). In postrenal AKI, rising intratubular/collecting-system hydrostatic pressure and dilatation restrict delivery and delay excretion of GSH-Au NCs (*66*), producing delayed parenchymal clearance with pelvic stasis and kidney enlargement.

These closely related mechanism-imaging links provide a reliable basis for Au-CT–based AKI classification and support a general workflow (Fig. 7B). In patients with suspected AKI, regardless of laboratory findings, assessing recent exposure to nephrotoxic drugs is preferred. If none or uncertain, patients can be treated with low-dose GSH-Au NCs (162.5 mg Au/kg). If there is a clear history of nephrotoxic drug use, an ultralow dose (32.5 mg Au/kg) can be administered. Although time-attenuation curves provide useful context, CT acquisition can be tailored to the clinical question. When renal perfusion assessment is important (e.g., suspected prerenal hypoperfusion or complex hemodynamics), an additional early corticomedullary/perfusion phase may be acquired, while a delayed scan at 10 to 30 min postinjection can provide stable intrarenal distribution patterns related to filtration and tubular handling. For selected high-risk patients or longitudinal follow-up, a single delayed acquisition (10 to 30 min) may serve as a time- and radiation-sparing option, rather than a replacement for multiphasic renal CT when comprehensive hemodynamic characterization is required. In addition, radiation dose-reduction techniques, like low-kilovolt protocols with automatic tube-current modulation and iterative/deep-learning reconstruction, can further lower radiation burden without sacrificing image quality (*67*, *68*). Accordingly, the radiologist reads the imaging pattern to provide a mechanism-based AKI diagnosis and classification. This streamlined, low-dose GSH-Au CT workflow enables early, mechanism-specific screening and longitudinal monitoring while minimizing patient burden.

This study also has several limitations. First, the experiments were confined to small animals, and a large-animal validation that better imitates human renal physiology is needed to calibrate dosing, kinetics, and readouts (*69*, *70*). Second, the disease scope was restricted to AKI, while imaging performance in chronic kidney disease, which affects roughly 10% of adults, remains to be determined, including fibrosis-predominant phenotypes and reduced-GFR states (*71*). Third, although short-term biochemistry and histology were benign after a single administration, formal toxicology programs (including immunogenicity, repeat-dose, and longer-term studies) will be required to define safety margins for translation (*72*). Repeat administration for serial monitoring also warrants evaluation of cumulative exposure and possible downstream consequences, including tubular obstruction or fibrosis. Last, we just evaluated the platform on conventional energy-integrating CT. Validation on spectral/dual-energy and photon-counting systems may enhance material separation and contrast-to-noise, which could enable further dose reductions (*30*). These gaps define clear priorities for translation and will guide future work.

In conclusion, we present a contrast-sparing CT framework using renal-clearable GSH-Au NCs that generates compartment-level maps of kidney dysfunction before serum indicators change. In naïve mice and three typical AKI models, low-dose GSH-Au NCs produced robust intrarenal contrast with rapid urinary clearance and revealed mechanism-specific imaging patterns, enabling a simple workflow for early AKI classification. This low-dose GSH-Au–enhanced CT platform offers an early, noninvasive, and spatially resolved method capable of detecting and localizing renal dysfunction in kidney diseases.

## MATERIALS AND METHODS

### Experimental design

These studies were designed to evaluate renal-clearable gold nanoclusters as a contrast-sparing CT agent for early spatial mapping of renal dysfunction and to assess their pharmacokinetics, renal clearance, and biosafety. In vivo studies were performed to determine probe performance in normal and diseased kidneys. Animals were randomly assigned to experimental groups at the beginning of each experiment. Healthy animals and renal dysfunction models were used to evaluate probe behavior under different physiological and pathological conditions. For image acquisition and quantitative analysis, investigators followed predefined criteria, and, where applicable, analysis was performed without knowledge of group allocation. Unless otherwise noted, in vitro and in vivo experiments were conducted with three independent replicates. No data were excluded from the analyses.

### Materials

HAuCl_4_∙3H_2_O was purchased from Heowns Co. Ltd. (Tianjin, China). l-reduced glutathione and cisplatin were acquired from Aladdin Biochemical Technology Co. Ltd. (Shanghai, China). Deionized water was bought from Wahaha Group Co. Ltd. (Hangzhou, China). Sodium hydroxide was available from Macklin Biotechnology Co. Ltd. (Shanghai, China). Isoflurane was supplied by RWD Life Science Co. Ltd. (Shenzhen, China). All chemicals and reagents were used as supplied, without additional purification.

### Synthesis of GSH-Au NCs

HAuCl_4_ (0.3 mmol) and 0.24 mmol of GSH (molar ratio of 1:0.8) were dissolved in 100 ml of deionized water and stirred at room temperature for 8 min in a 500-ml single-neck flask, during which the solution’s color changed from yellow to pale yellow or nearly colorless. The mixture was then heated in an oil bath at 90°C for 35 min, resulting in a clear red-brown solution. After cooling to room temperature, large aggregates were removed by filtration through a 220-μm aqueous phase membrane. The pH was subsequently adjusted to ~3 with sodium hydroxide, and ethanol (2:1 in volume, ethanol to solution) was added to precipitate the Au NCs, which were then centrifuged at 10,000 rpm for 5 min. The precipitate, redissolved in PBS (pH 7.4), was purified by dialysis against deionized water for 48 hours. Last, the GSH-Au NCs were freeze-dried to obtain a solid and stored in a refrigerator at 4°C.

### Characterization of GSH-Au NCs

The morphology and size of GSH-Au NCs were characterized by a JEOL JEM 2100F transmission electron microscope (Japan) with an acceleration voltage of 200 kV. The hydrodynamic size distribution and zeta potentials were measured using a Malvern Zeta-sizer (Nano Series ZS, UK). FTIR spectra in the range of 400 to 4000 cm^−1^ were recorded using a Nicolet iS10 spectrometer (Nicolet, USA), with dried pure potassium bromide used as the background. UV absorption and fluorescence spectra were recorded using a UV-3600 Plus UV-vis-NIR spectrophotometer (Shimadzu, Japan) and an F7000 fluorescence spectrophotometer (Hitachi, Japan), respectively. The Au concentrations were quantified using inductively coupled plasma optical emission spectrometry (ICP-OES; Agilent 5800, USA) and ICP-MS (Agilent 8900, USA). Serum liver and kidney function indices were assessed using an automated biochemical analyzer (BS-430, Mindray, China).

### Gel electrophoresis analysis of nanocluster-protein interactions

Four groups were prepared: a protein marker, GSH-Au NCs alone, BSA alone, and GSH-Au NCs incubated with BSA. Each sample was mixed with an appropriate volume of loading buffer. Nanocluster-protein binding reactions were performed in 1.5-ml microcentrifuge tubes by directly mixing nanocluster suspensions in PBS (25 mg/ml) with BSA (0.5 mg/ml), followed by incubation at 37°C for 30 min. The mixtures were then loaded onto 1% agarose gels. Electrophoresis was carried out in 1× tris-borate-EDTA buffer at a constant voltage of 80 V for 40 to 50 min. The gel was stained with CBB-250 to visualize the protein bands, and, then, gel images were acquired using a digital camera.

### Colloid stability of GSH-Au NCs in vitro

GSH-Au NCs (1 mg/ml) were dispersed in various media, including deionized water, normal saline, PBS (10 mM, pH 7.4), fetal bovine serum (Lanzhou Bailing Biotechnology Co. Ltd.), and Dulbecco’s modified Eagle’s medium (Thermo Scientific Co. Ltd.). Digital photographs of the dispersions were captured at designated intervals over an 18-day period to systematically evaluate changes in optical clarity and transparency.

### CT imaging of GSH-Au NCs in vitro

The CT imaging performance of GSH-Au NCs was assessed using a micro-CT system (IRIS, Germany) and a dual-source CT scanner (SOMATOM Force, Siemens Healthineers, Germany). GSH-Au NCs and Ioversol solutions were prepared with Au or iodine concentrations of 0, 10, 25, 50, 100, and 200 mM for phantom imaging. Micro-CT scans were performed with a slice thickness of 0.1 mm, adaptive tube current, and a fixed tube voltage of 50 kV. Conventional CT scans were acquired with a field of view (FOV) of 256 mm by 256 mm, a slice thickness of 0.5 mm, adaptive tube current, and x-ray tube voltages ranging from 70 to 150 kV. Spectral CT scans were performed under the same FOV and slice thickness, with a tube voltage of 90/150 Sn (equivalent to conventional 120 kV). Spectral images were reconstructed and analyzed across a monochromatic x-ray energy range of 40 to 190 keV. Data processing was performed using Synovia software (Siemens Healthineers, Germany), and the HU values were used to plot concentration-HU curves.

### Hemolysis assay

Fresh blood (1 ml) was collected from the orbital sinus of C57BL/6 mice and transferred to anticoagulant tubes. The sample was diluted with 1.5 ml of PBS and left at room temperature for 1 hour. Red blood cells were isolated by centrifugation at 3000 rpm for 5 min and washed three times with PBS. The RBCs were then resuspended in 4 ml of PBS and incubated with GSH-Au NCs at concentrations of 10, 20, 40, 80, and 100 mg/liter. PBS and distilled water served as negative and positive controls, respectively. After 2 hours of incubation at room temperature, photographs were taken, and the supernatant was transferred to a 96-well plate. Absorbance at 541 nm was measured using a microplate reader. The hemolysis percentage (HP) was calculated using the equation: HP = (sample − A_negative)/(A_positive − A_negative) × 100%.

### Experimental animal

The experimental animals used in this study included Balb/c mice (female) and C57BL/6 mice (male), 8 to 10 weeks, with an average body weight of ~20 g. The mice were obtained from Beijing SPF Biotechnology Co. Ltd. [SCXK (Beijing) 2019-0010]. All animal experiments were conducted in accordance with protocols approved by the Laboratory Animal Ethics Committee of Tianjin Medical University General Hospital (IRB2022-DW-76). For in vivo studies, a group size of three animals was selected on the basis of pilot experiments, our prior experience with this animal model, and ethical considerations to minimize animal use while allowing preliminary assessment of treatment effects.

### Biocompatibility evaluation in vivo

The biocompatibility of GSH-Au NCs was assessed in vivo through monitoring of body weight, blood biochemical analyses, and histopathological examination of major organs (heart, liver, spleen, lungs, and kidneys). Balb/c mice in the treatment group (*n* = 3) received GSH-Au NCs via tail vein injection at a dose of 325 mg Au/kg body weight, while mice in the control group (*n* = 3) were administered an equal volume of PBS. Body weights were recorded over a 14-day observation period. Serum samples were collected at predetermined time points (1 day, 7 days, and 14 days postinjection) for subsequent blood biochemical analyses. The measured parameters included hepatic function markers [alanine aminotransferase (ALT), aspartate aminotransferase (AST), albumin (ALB), and total protein (TP)] as well as renal function markers [serum creatinine (CREA), blood urea nitrogen (UREA), and uric acid (UA)]. Simultaneously, major organs (heart, liver, spleen, lungs, and kidneys) were dissected from mice in each group (control, day 1, day 7, and day 14), fixed in 4% formaldehyde solution, and subjected to H&E staining for histological evaluation.

### Pharmacokinetics in vivo

To investigate the pharmacokinetic behavior of GSH-Au NCs in vivo, Balb/c mice (*n* = 3) were administered a single dose of GSH-Au NCs (325 mg Au/kg) via tail vein injection. Blood samples were collected at designated time points postinjection (3 min, 10 min, 15 min, 30 min, 1 hour, and 2 hours). Each blood sample was digested in 68% concentrated nitric acid, followed by appropriate dilution with deionized water. The Au concentration in each sample was then quantitatively determined using ICP-OES. Based on the temporal Au concentrations measured in blood, the circulation half-life of the GSH-Au NCs was calculated.

### Biodistribution in vivo

Balb/c mice were intravenously administered GSH-Au NCs via tail vein injection at a dose of 325 mg Au/kg. At designated time points postadministration (10 min, 1 day, 7 days, and 14 days), major organs—including the heart, liver, spleen, lungs, and kidneys—were harvested and weighed (*n* = 3 per time point). The collected organs were digested in 68% concentrated nitric acid and subsequently diluted with deionized water. The Au content in each organ was then quantified using ICP-OES.

### Excretory behavior of GSH-Au NCs in vivo

The in vivo excretory fate of GSH-Au NCs (325 mg Au/kg) was systematically investigated using CT and NIR fluorescence imaging. Healthy Balb/c mice were imaged using an IRIS micro-CT scanner. Complementary NIR fluorescence imaging was used to monitor the biodistribution and clearance of GSH-Au NCs. Micro-CT scans were acquired prior to tail vein injection and at multiple postinjection time points (immediately, 5 min, 10 min, 15 min, 30 min, 1 hour, 2 hours, and 24 hours). Urine samples were collected before injection and at 30 min and 24 hours postinjection for CT analysis. Imaging parameters were set as follows: slice thickness, 0.1 mm; and tube voltage, 50 kV. CT attenuation values were quantified in the heart, liver, kidneys, and bladder. To assess renal and systemic excretion of GSH-Au NCs, multiplanar reconstruction was performed using the RadiAnt DICOM Viewer. ROIs were defined in the heart, liver, renal cortex, OM, IM, renal pelvis, and bladder (*n* = 3), and CT values with SD were recorded. ROIs were maintained at a consistent size and measured at least three times to calculate average values. NIR fluorescence imaging was performed with an excitation wavelength of λ_ex = 710 nm, an emission range of λ_em = 810 to 875 nm, an exposure time of 1 s, a pixel binning of 2, and an aperture of f/1. Whole-body fluorescence signals were recorded at the same points as CT imaging (immediately, 5 min, 10 min, 15 min, 30 min, 1 hour, 24 hours, and 48 hours). At 48 hours postinjection, major organs (heart, liver, spleen, lungs, and kidneys) were harvested for ex vivo imaging. Fluorescence intensities were quantified using Living Image software (PerkinElmer, USA). To determine the renal clearance efficiency of GSH-Au NCs, normal mice were intravenously injected with GSH-Au NCs (325 mg Au/kg). All urine samples excreted within 2 hours postinjection were collected, and the gold content in the urine was quantified by ICP-OES to estimate the renal clearance efficiency.

### Imaging of prerenal AKI in vivo

Balb/c mice were anesthetized with isoflurane, and a left dorsal incision was made to expose and ligate the lower branches of the renal artery using 3-0 absorbable sutures. Approximately 10 min after ligation, the affected region developed a dark-purple coloration, confirming successful model establishment. The incision was then closed and disinfected. Four hours postsurgery, micro-CT and fluorescence imaging were performed.

For CT studies, mice received intravenous injections of GSH-Au NCs (162.5 or 325 mg Au/kg) or equimolar doses of Ioversol (104.5 or 209.4 mg I/kg) (*n* = 3 per group). Micro-CT scans were acquired before injection and immediately, 5 min, 10 min, 15 min, 30 min, 1 hour, and 2 hours postinjection. Raw datasets were reconstructed using RadiAnt DICOM Viewer, with ROIs delineated in normal and ischemic regions of the left renal parenchyma. CT values were recorded to calculate the DS and RRF, and volumetric reconstruction of renal regions was performed. DS = CT value (normal region)/CT value (injured region). RRF = 100% × CT value (ROI)/CT value (same region on contralateral kidney). For fluorescence imaging, mice were injected with GSH-Au NCs (325 mg Au/kg, *n* = 3), and whole-body images were acquired at the same pre- and postinjection time points. After in vivo imaging, the mice were treated by blood collection via the orbital sinus and excision of both kidneys for histopathological analysis.

### Imaging of intrarenal AKI in vivo

C57BL/6 mice were administered cisplatin (15 mg/kg, intraperitoneally, dissolved in 10 mM pH 7.4 PBS), and body weight was monitored daily, with weight loss serving as an indicator of successful AKI induction. On 24 and 72 hours postinjection, mice were randomly assigned to three cohorts (*n* = 3 per group): two received intravenous GSH-Au NCs (32.5 and 162.5 mg Au/kg), and the other one was injected with Ioversol at an equimolar iodine dose (20.9 mg I/kg, corresponding to the low-dose GSH-Au NCs group). Micro-CT scans were performed before injection and immediately, 5 min, 10 min, 15 min, 30 min, 1 hour, 2 hours, and 24 hours after administration. To assess the diagnostic performance of GSH-Au NCs at different doses and severities of intrarenal AKI, ROIs were delineated in the cortex, OM, IM, and renal pelvis of both kidneys, and CT values were recorded to analyze temporal dynamics. To confirm the renal distribution of nanoparticles, in vivo imaging of another group of model mice (*n* = 3) was terminated at 30 min postinjection. Blood was then collected via the orbital sinus, and both kidneys were excised for ex vivo micro-CT scanning and histopathological analysis.

To further evaluate the impact of x-ray spectral differences between micro-CT (50 kV) and clinical CT (80 to 120 kV), in vivo validation experiments were conducted on a clinical CT scanner (SOMATOM Force, Siemens Healthineers, Germany). At 24 hours after cisplatin administration, mice received an intravenous injection of GSH-Au NCs (32.5 mg Au/kg) via the tail vein. Conventional CT scans were acquired before contrast administration and at 1, 5, 10, 15, and 30 min, as well as 1 and 2 hours postinjection. Conventional CT imaging was performed with a slice thickness of 0.5 mm, adaptive tube current, and tube voltages of 80, 100, and 120 kV. For quantitative image analysis, raw data were analyzed using RadiAnt DICOM Viewer. ROIs were delineated in the renal medulla before and after contrast administration to obtain CT attenuation values. Image noise was defined as the SD of CT values measured in a homogeneous background region. ROI areas were kept constant across measurements, and each region was measured at least four times to obtain mean CT values. The CNR was calculated as: CNR = (CT_post_ − CT_pre_)/|SD_background_|.

### Imaging of postrenal AKI in vivo

C57BL/6 mice were anesthetized with a mixture of oxygen and isoflurane, and a one-sided flank incision was made to expose the ureter, which was then completely ligated with 3-0 absorbable sutures. The incision was subsequently closed and disinfected. At 12 and 72 hours postsurgery, mice (*n* = 3 per group) received intravenous injections of GSH-Au NCs (162.5 mg Au/kg), followed by micro-CT scans performed prior to injection and at 0, 5, 10, 15, and 30 min, as well as 1, 2, and 24 hours postinjection. To evaluate the diagnostic performance of GSH-Au NCs in postrenal AKI of varying severity, raw CT datasets were reconstructed using multiplanar reformation. ROIs were delineated in the cortex, medulla, and renal pelvis of both kidneys, and CT values were recorded to compare differences between the obstructed and contralateral kidneys. To quantify morphological alterations induced by ureteral obstruction, the longest renal axis (*a*) and the maximum perpendicular short axis (*b*) were measured, and their product (*a* × *b*) was used as the maximum cross-sectional area to assess inter-kidney differences. The in vivo imaging of another group of model mice (*n* = 3) was terminated at 30 min postinjection, followed by blood collection via the orbital sinus and excision of both kidneys for ex vivo micro-CT and histopathological analysis.

### Statistical analysis

All data were analyzed using GraphPad Prism 9.5.1 (CA, USA). Quantitative data are presented as means ± SD. For comparisons among three or more groups, one-way analysis of variance (ANOVA) was performed. For comparisons between two groups, a *t* test was used. Statistical significance levels were defined as follows: not significant (n.s.), *P* > 0.05; *P < 0.05; **P < 0.01; ***P < 0.001; ****P < 0.0001.
