# Supplementary material for: Contrast-sparing CT using renal-clearable gold nanoclusters for early spatial mapping of renal dysfunction
Source: Sci Adv. 2026 May 29;12(22):eaec0972. doi: 10.1126/sciadv.aec0972 (PMC13220870; doi:10.1126/sciadv.aec0972)
Supplement: Supplementary file 1 — Figs. S1 to S13 [file sciadv.aec0972_sm.pdf]

Science Advances
AAAS

Supplementary Materials for

Contrast-sparing CT using renal-clearable gold nanoclusters for early spatial mapping of renal dysfunction

Jinbin Pan *et al.*

Corresponding author: Peter Caravan, pcaravan@mgh.harvard.edu; Shao-Kai Sun, shaokaisun@tmu.edu.cn

Sci. Adv. **12**, eaec0972 (2026)
DOI: 10.1126/sciadv.aec0972

This PDF file includes:

Figs. S1 to S13

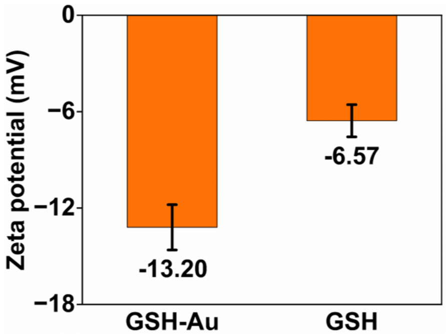

Fig. S1. Zeta potentials of GSH-Au NCs and GSH.

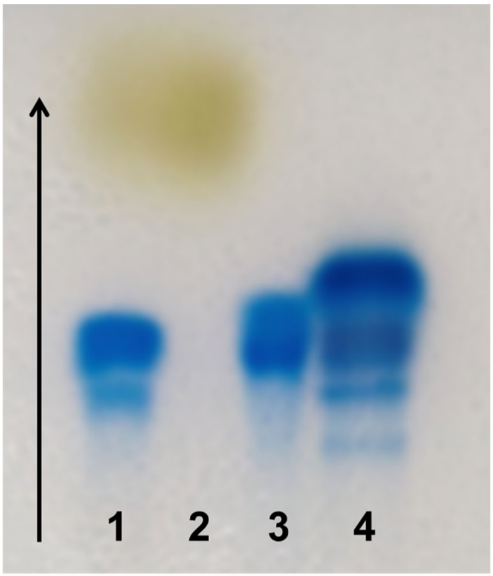

Fig. S2. Agarose gel electrophoresis of GSH-Au NCs with or without incubation with BSA. GSH-Au NCs were incubated in the presence (well 1) or absence (well 2) of CBB250-stained BSA (0.5 mg/mL) at 37 °C for 30 min. The blue band in well 3 = CBB250-stained BSA. The blue band in well 4 = CBB250-stained protein marker.

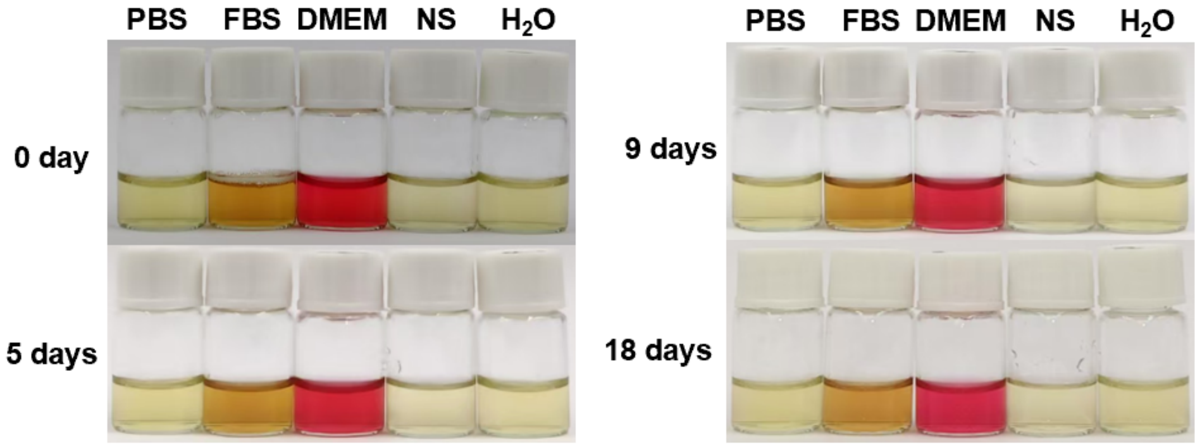

Fig. S3. Photographs of GSH-Au NCs (1 mg/mL) dispersed in different media for 18 days. PBS, phosphate buffer solution; FBS, fetal bovine serum; DMEM, Dulbecco's modified Eagle medium; NS, normal saline.

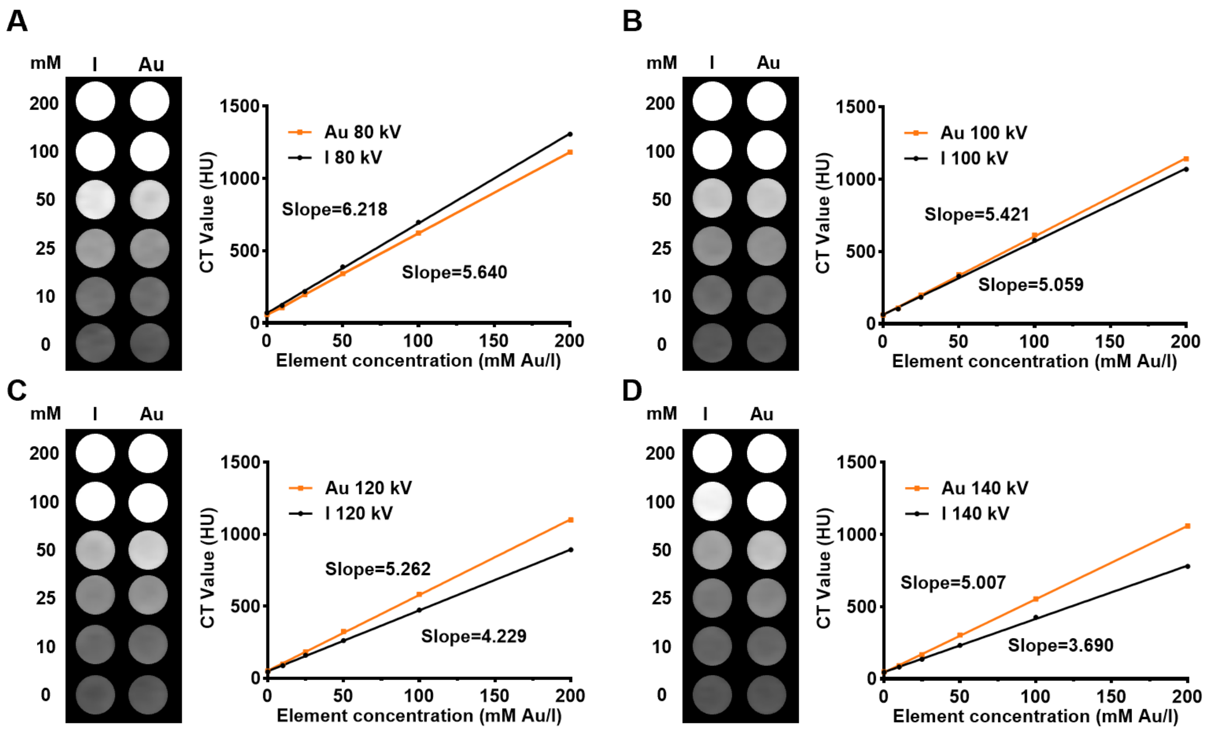

Fig. S4. Attenuation-concentration plots of GSH-Au NCs and Ioversol acquired at different tube voltages (A, 80 kV; B, 100 kV; C,120 kV; D, 140 kV) with corresponding phantom grayscale images.

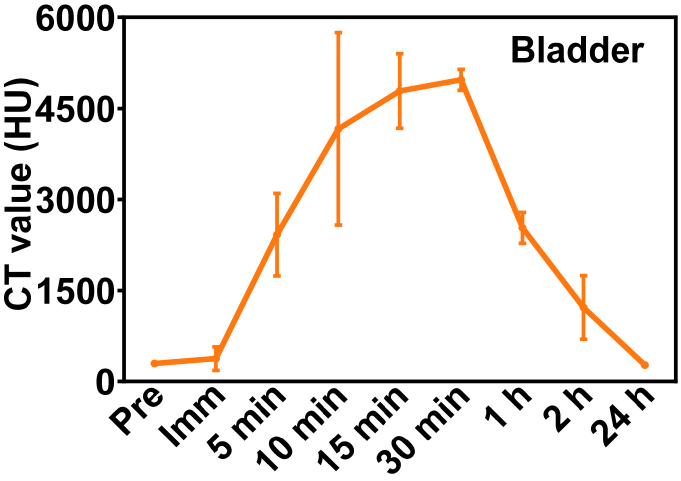

Fig. S5. Time-attenuation curves of bladder after administration of GSH-Au NCs (325 mg Au/kg). *N* = 3 per time point.

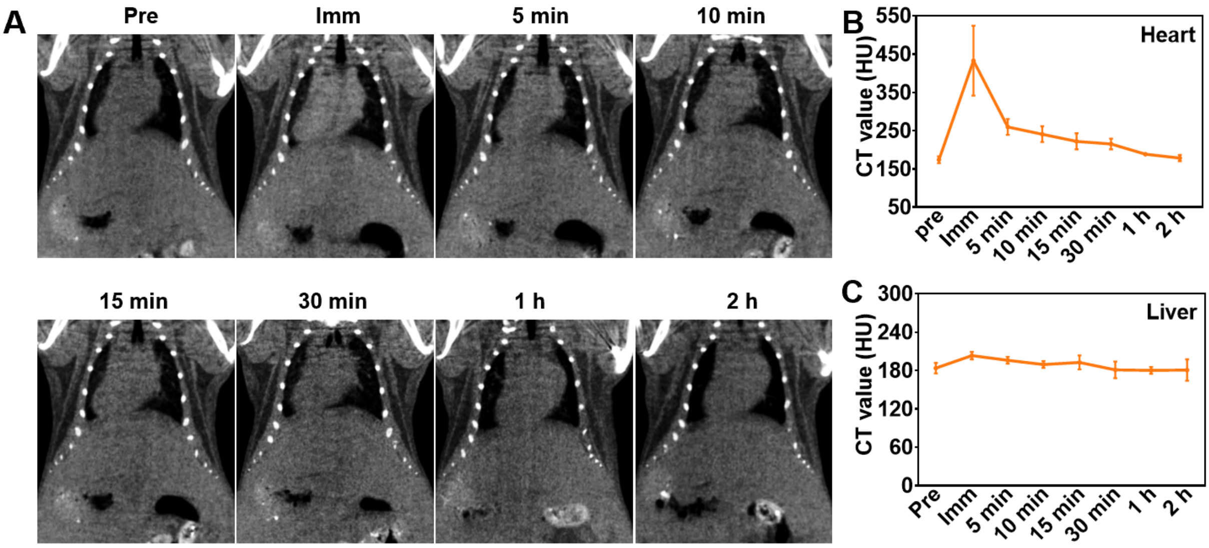

Fig. S6. In vivo CT imaging of renal-clearable GSH-Au NCs (325 mg Au/kg). (**A**) CT images of the heart and liver before and at different time points after injection. (**B, C**) Corresponding time-attenuation curves of heart (**B**) and liver (**C**). *N* = 3 at each time point.

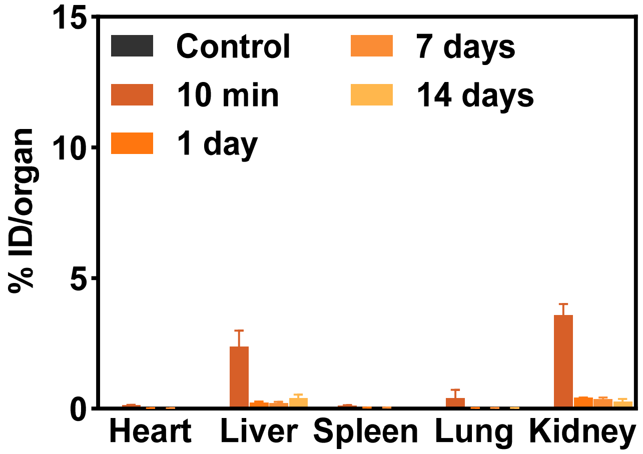

Fig. S7. Biodistribution of Au showed as total %ID per organ at 10 min, 1 day, 7 days, and 14 days post-injection and in non-injected animals (control). *N* = 3 per time point.

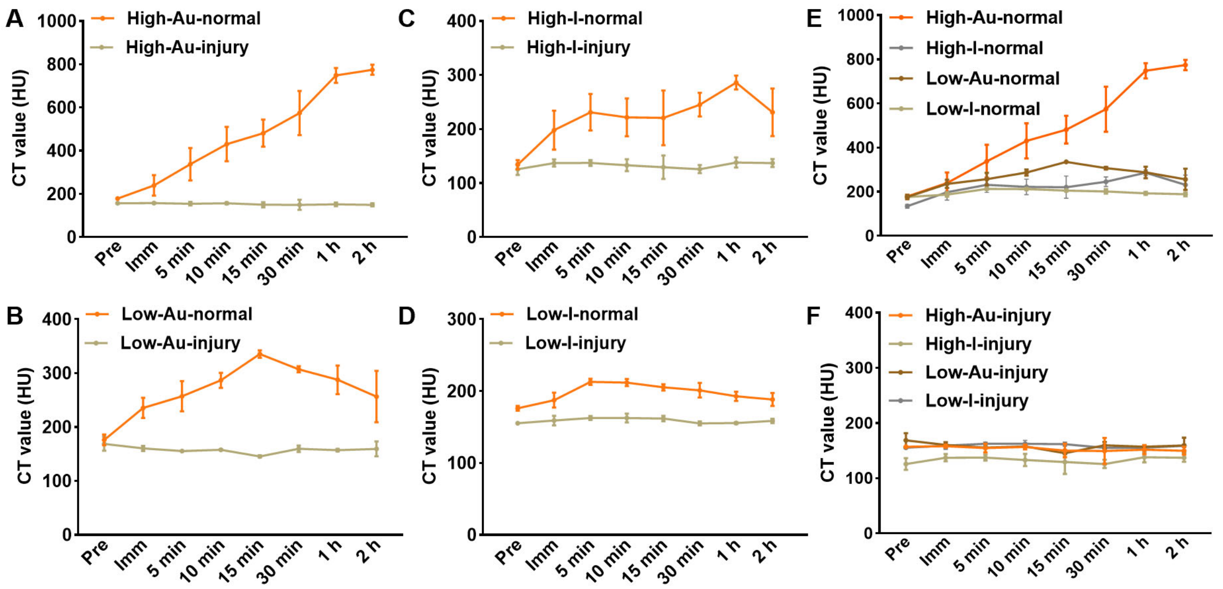

Fig. S8. Time-attenuation curves from normal and injured renal parenchyma in mice (*N* = 3 per group) given different contrast agents. (**A**) 325 mg Au/kg (High-Au) GSH-Au NCs, (**B**) 162.5 mg Au/kg (Low-Au) GSH-Au NCs, (**C**) Ioversol at a molar-equivalent iodine dose to 325 mg Au/kg, 209 mg I/kg (High-I). (**D**) Ioversol at a molar-equivalent iodine dose to 162.5 mg Au/kg, 104.5 mg I/kg (Low-I). (**E, F**) Time-attenuation curves of normal (**E**) and injured (**F**) renal parenchyma with given contrast agents.

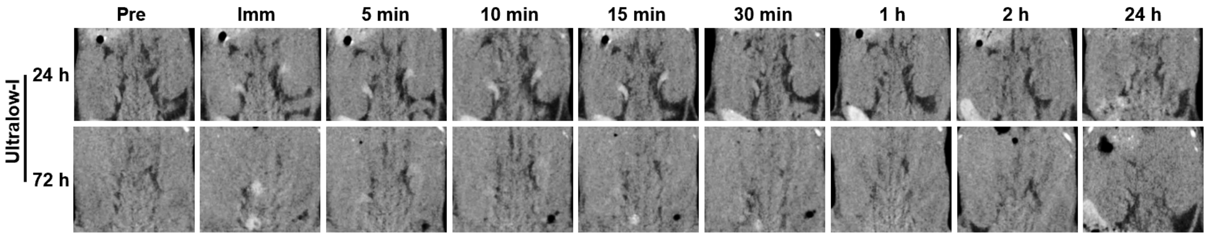

Fig. S9. Serial contrast-enhanced CT kidney imaging acquired under ultralow dose Ioversol (20.9 mg I/kg, equimolar to 32.5 mg Au/kg GSH-Au NCs). Scans were performed in naïve mice and in cisplatin-treated mice at 24 h and 72 h post-cisplatin.

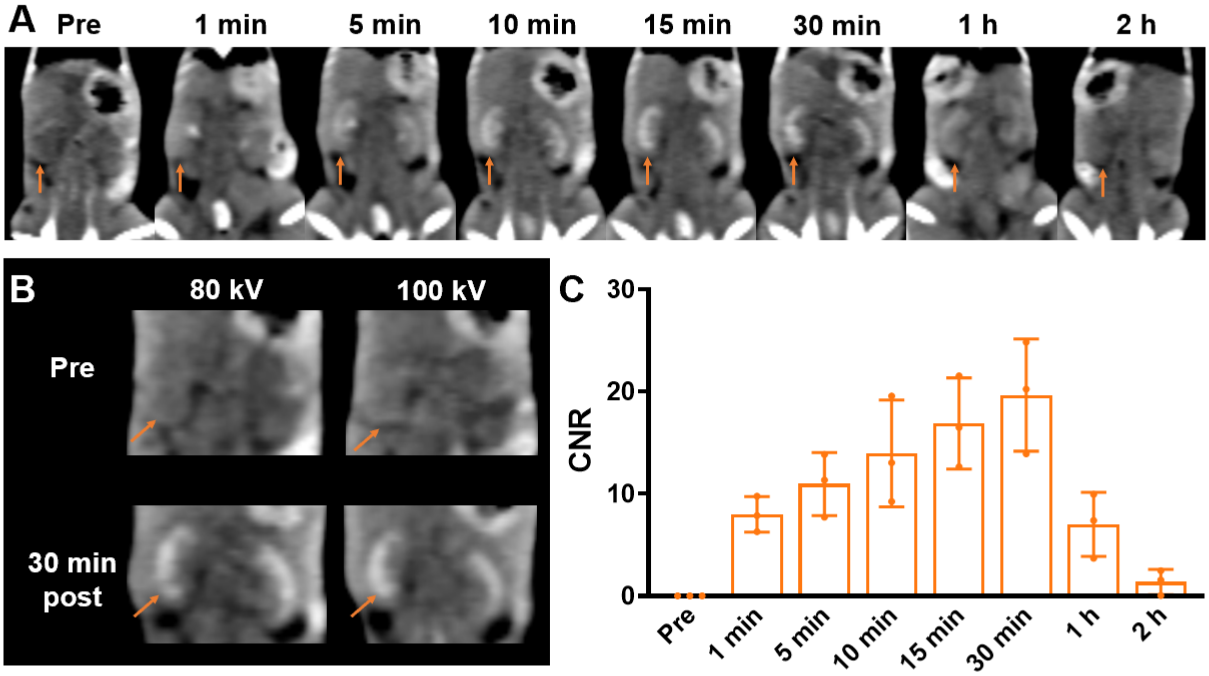

Fig. S10. Clinical CT validation of ultralow-dose GSH-Au NCs for early cisplatin-AKI imaging. (A) Representative coronal clinical CT images acquired before (Pre) and at 1, 5, 10, 15, and 30 min, 1 h, and 2 h after intravenous injection of GSH-Au NCs (32.5 mg Au/kg) in the cisplatin-AKI (24 h) mouse model at 120 kV (0.5-mm slice thickness). Orange arrows indicate the kidney/outer-medullary enhancement. (B) Representative coronal images acquired at 80 and 100 kV before injection (Pre) and at 30 min post-injection, showing a similar enhancement pattern (orange arrows) under different clinical tube voltages. (C) Time course of outer-medullary contrast-to-noise ratio (CNR) measured on clinical CT (120 kV) relative to baseline. Data are shown as mean ± SD (n = 3).

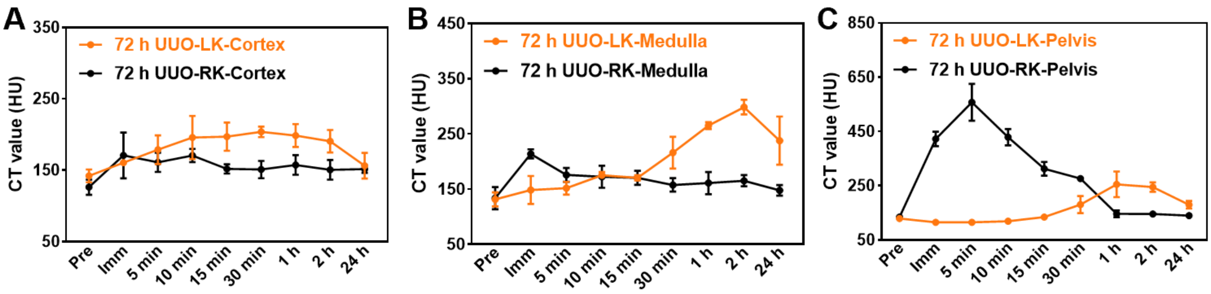

Fig. S11. Compartment-based time-attenuation curves (cortex, medulla, pelvis) after contrast injection in 72 h UUO mice. LK, left kidney (obstructed); RK, right kidney (non-obstructed). $N = 3$ at each time point.

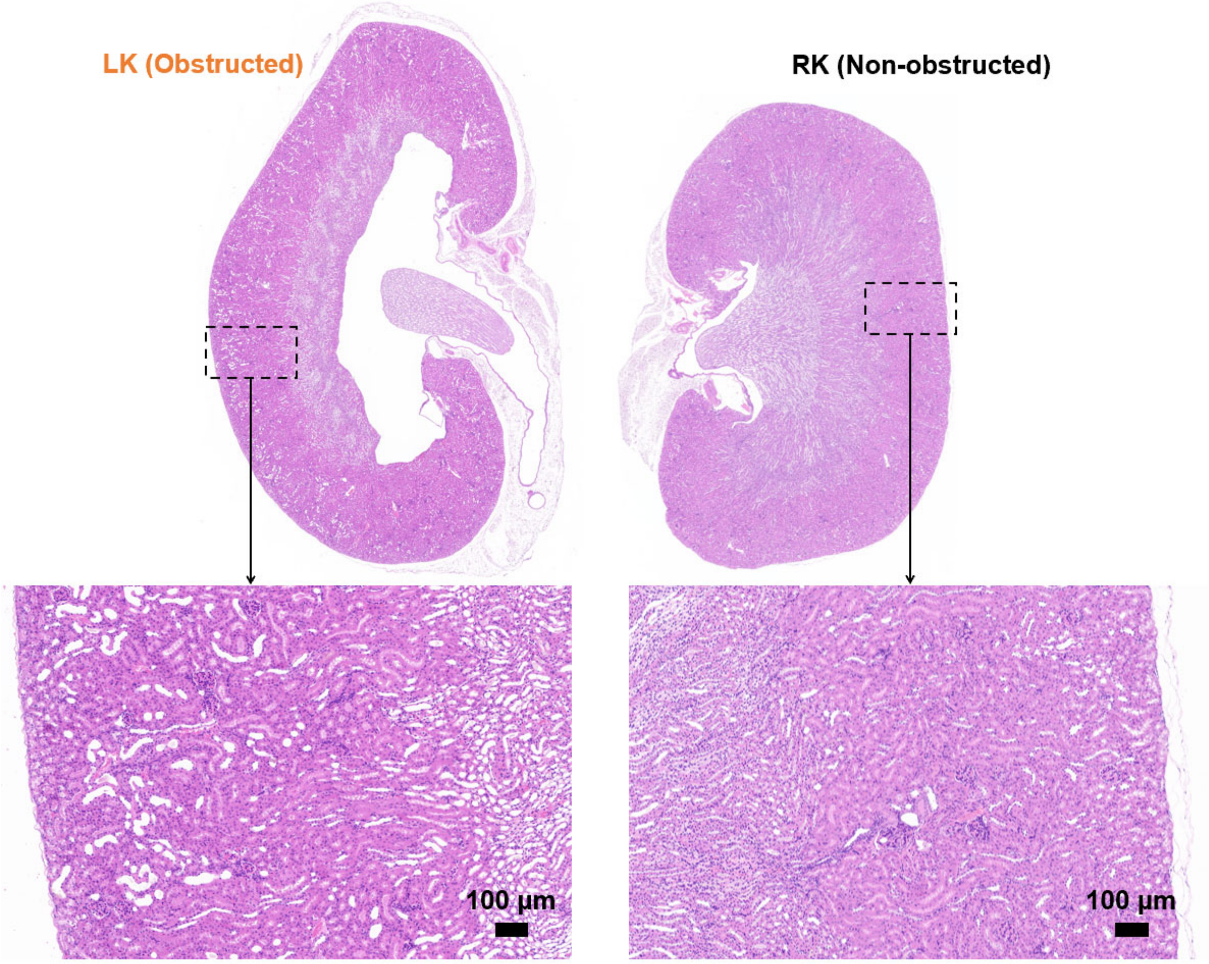

Fig. S12. H&E staining of bilateral kidneys in a 12 h UUO model after intravenous injection of GSH-Au NCs (162.5 mg Au/kg). The obstructed kidney (LK) showed significantly dilated renal tubules compared to the contralateral non-obstructed kidney (RK).

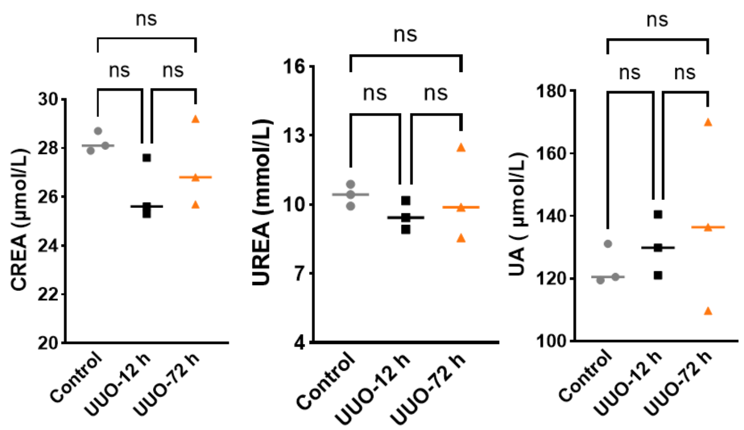

Fig. S13. Post-imaging serum biochemistry (creatinine, urea, uric acid) of naïve mice (control) and UUO mice for different obstruction times (12 h and 72 h). $N = 3$ per group; ns, $P > 0.05$.
